# Phytochemical Analysis and Dermo-Cosmetic Evaluation of *Cymbidium* sp. (Orchidaceae) Cultivation By-Products

**DOI:** 10.3390/antiox11010101

**Published:** 2021-12-31

**Authors:** Evangelos Axiotis, Apostolis Angelis, Lemonia Antoniadi, Eleftherios A. Petrakis, Leandros A. Skaltsounis

**Affiliations:** 1 Department of Pharmacy, Division of Pharmacognosy and Natural Products Chemistry, National and Kapodistrian University of Athens, Panepistimiopolis Zografou, 15771 Athens, Greece; axiotisevan@pharm.uoa.gr (E.A.); monikaant@pharm.uoa.gr (L.A.); epetrakis@pharm.uoa.gr (E.A.P.); 2 Natural Products Research Center “NatProAegean”, Gera, 81106 Lesvos, Greece

**Keywords:** Orchidaceae, *Cymbidium* sp., antioxidant activity, tyrosinase, elastase, collagenase, phytochemical analysis, NMR, by-products, North Aegean

## Abstract

*Cymbidium* is one of the most popular genera in Orchidaceae family, commercialized either as loose flowers or as potted plants in floriculture worldwide. The non-marketable parts are typically discarded (e.g., unsuitable flowers, leaves, pseudobulbs, roots), generating an enormous quantity of unutilized biomass. The above by-products were studied through phytochemical analysis and investigated for their dermo-cosmetic potential. The initial antioxidant, anti-tyrosinase, anti-elastase, and anti-collagenase assays of the total extracts indicated that the pseudobulb and root ethyl acetate extracts were the most potent. Those extracts were then submitted to chromatographic separation leading to the isolation of 16 secondary metabolites (four phenanthrenes, three 1,4-phenanthrenquinones, three dibenzyls, two phenolic acid derivatives, two sterols, one dehydrodiconiferyl alcohol derivative, and one simple phenolic compound), including 6-hydroxy-5,7-dimethoxy-1,4-phenanthrenequinone (cymbisamoquinone), which was identified as a new natural product. In parallel, 48 metabolites were identified by UPLC-HRMS analysis of the extracts. The biological evaluation of the isolated compounds revealed that gigantol and tristin present important anti-tyrosinase activity, while bulbophyllanthrin, 3-hydroxy-2,4,7-trimethoxy-phenanthrene, marylaurencinol A, 5-hydroxy-2-methoxy-1,4-phenanthrenequinone, and ephemeranthroquinone B show dose-dependent anti-collagenase activity. In contrast to isolated metabolites, which may act selectively on specific enzymes, the initial total extracts exhibited inhibitory activity against tyrosinase, elastase, and collagenase enzymes, thus showing better prospects for use in dermo-cosmetic formulations.

## 1. Introduction

Orchidaceae is the largest botanical family of flowering plants with a total number of species varying between 25,000 and 35,000 [1,2]. According to “The Plant List” [3], this family currently contains 27,801 species divided into 925 genera, and due to its remarkable capacity for adaptation, it is present in all regions of the world in the wild. The main reason lies in the deterioration of their native ecosystems as a consequence of human activities [2].

Τhe medicinal uses of various orchid species are well known within so-called traditional medicine throughout the world. Orchids were first cultivated and described for medicinal use in China. A total of 365 herbs, including several orchids, are listed in the Chinese Materia Medica [4]. In the western hemisphere, Theophrastus made the first description of this species, naming them “Orchids” from the Greek word “Orchis”, due to its resemblance to a pair of testicles [4]. Dioscorides, in De Materia Medica, included two terrestrial orchids and described the medicinal use of the tubers according to their resemblance to parts of human anatomy (shape and color) [5]. The most represented genera of medicinal orchids are *Calanthe*, *Coelogyne*, *Cymbidium*, *Cypipedium*, *Dendrobium*, *Ephemerantha*, *Eria*, *Galeola*, *Gastrodia*, *Gymnadenia*, *Habenaria*, *Ludisia*, *Luisia*, *Nevilia*, and *Thunia* [6]. Alkaloids, bibenzyl derivatives, flavonoids, carotenoids, phenanthrenes, phenanthropyrans, stilbenes, anthocyanins, glycosides, sterols, and terpenoids present the most important phytochemical classes among the Orchidaceae species that are associated with pharmacological activities [1,7,8,9,10,11,12].

The genus *Cymbidium* comprises approximately 64 species and numerous natural hybrids and is one of the most commercially popular orchids along with *Phalaenopsis* spp., both of which are utilized as cut flowers and potted plants in floriculture worldwide. Modern *Cymbidium* hybrids are traced back to fewer than ten species, mostly growing in the higher elevations of the mountainous backbone of Asia [13]. Geographic distribution extends from the northwestern Himalayas to Japan, then to Vietnam and Malesia and to northern and eastern Australia. In 1924, Schlechter [14] proposed a major revision of *Cymbidium* based on the most recent information on the species’ morphological and anatomical characteristics. The plant is generally characterized by its composed flowers, which exhibit a wide range of colors [15].

**Figure 1:**
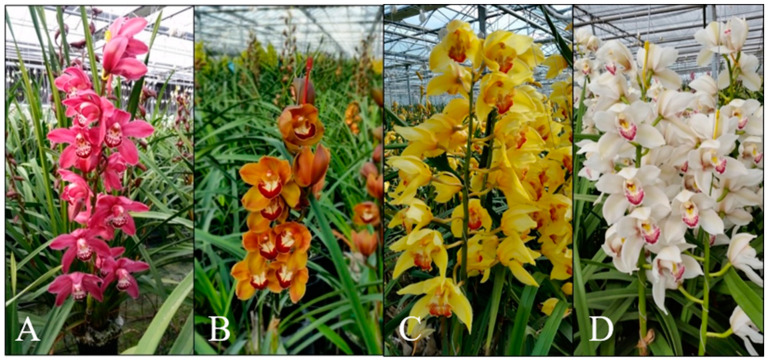
*Cymbidium* sp. cultivated orchids in Samos Island. The four different *Cymbidium* hybrids are registered in the Royal Horticultural Society (International Registration Authority for Orchid) as follows: (**A**) *Cymbidium* Tethys; (**B**) *Cymbidium* Jazz festival; (**C**) *Cymbidium* Tracey Reddaway; (**D**) *Cymbidium* Thomas Starzl.

Anthocyanin accumulation (cyanidin 3-*O*-glucoside, cyanidin 3-*O*-rutinoside, and cyanidin 3-*O*-(6″-malonylglucoside)) is responsible for red, pink, and purple color, whereas carotenoids and chlorophylls are responsible for yellow and green colors, respectively. Furthermore, short and stout pseudobulbs are present, with the flower spikes developing from the base of the pseudobulb. The leaves are long, ribbon shaped, soft, and lanceolate. In Figure 1, diverse flowers of *Cymbidium* are presented, with different color variations as a result of hybridization within this genus.

Various research studies have demonstrated the antimicrobial, anti-inflammatory, antinoceptive, and antioxidant effects of plants from the genus *Cymbidium* [16,17,18,19,20], as well the use of their volatile ingredients for cosmetic uses [10]. Although several phytochemical studies have been reported in the literature on different species of *Cymbidium*, focused mainly on the identification of phenanthrene derivatives, dibenzyls, alkaloids, and triterpene glycosides [11,21,22,23], a targeted phytochemical analysis aimed at biological activity for dermo-cosmetic use of *Cymbidium* sp. has not been previously reported.

The main use of *Cymbidium* sp. hybrids, worldwide, is for ornamental purposes. The non-marketable parts are usually discarded (e.g., unsuitable flowers, leaves, pseudobulbs, and roots), thus generating an enormous quantity of unutilized biomass. In the current study, the above by-products were evaluated for their potential as dermo-cosmetic agents, with a view to contribute to the agri-food economy of the islands of the North Aegean Sea. In this context, we proceeded with the extraction of the raw material and the study of the recovered extracts for their antioxidant capacity and anti-aging activity. The ethyl acetate extracts of the pseudobulbs and roots were the most potent, and thus their chemical characterization followed. The isolated compounds were then structurally elucidated and investigated for their bioactivity, highlighting the prospects for these orchid by-products.

## 2. Materials and Methods

### 2.1. Reagents and Materials

For extraction, analytical grade solvents, Dichloromethane (DCM), Ethyl Acetate (EtOAc), and Ethanol (EtOH) were purchased from Carlo Erba Reactifs SDS (Val de Reuil, France). The aqueous solutions were prepared using deionized water. For TLC analysis, solvents were obtained from Fisher Scientific, while Sulphuric Acid (H_2_SO_4_) and Vanillin standard (98%) were purchased from Sigma-Aldrich (Steinheim, Germany). For HPLC analysis, Methanol (MeOH), Acetonitrile (can), Water, and Acetic Acid were obtained from Fisher Scientific (Leicestershire, UK). ACN and Formic Acid used for LC–MS analysis were of LC–MS grade (Fisher Scientific, Loughborough, UK), while ultrapure water was obtained from a Milli-Q^®^ purification system (Merck Millipore, Darmstadt, Germany). Dimethyl sulfoxide (DMSO), DPPH (2,2-diphenyl-1-picrylhydrazyl), Gallic Acid used for DPPH Radical Scavenging assay, as well all reagents used for the three enzymatic assays, were purchased from Sigma-Aldrich. Kojic Acid (purity 99%), Elastatinal, and Phosphoramidon were used as the positive control for anti-tyrosinase, anti-elastase, and anti-collagenase assays, respectively, and were also purchased from Sigma-Aldrich.

### 2.2. Plant Material

The whole plant of *Cymbidium* was kindly provided by the “Garoufalis” greenhouse facilities on the Greek island of Samos (North Aegean Sea) in 2020. Its annual production amounts to more than 100,000 pots of 80 different varieties of *Cymbidium* sp., specializing in the cultivation of two basic types: ‘standard’ and ‘mini’. Four different hybrids of *Cymbidium* sp. were provided, as shown in Figure 1. The hybrids are registered in the Royal Horticultural Society (International Registration Authority for Orchid) as *Cymbidium* Tethys (Figure 1A), *Cymbidium* Jazz Festival (Figure 1B), *Cymbidium* Tracey Reddaway (Figure 1C), and *Cymbidium* Thomas Starlz (Figure 1D). The voucher specimens (SAM110) have been deposited at the herbarium of the Faculty of Pharmacy, Department of Pharmacognosy and Natural Products Chemistry, National and Kapodistrian University of Athens, Greece. Flowers, leaves, pseudobulbs, and roots were separated and dried at room temperature. The dried material was then powdered in a mill, and portions thereof were subjected to ultrasound-assisted extraction (UAE).

### 2.3. Plant Extraction

Quantities of 400 g of powdered, shade-dried plant material of *Cymbidium* flowers, leaves, pseudobulbs, and roots were separately extracted successively with dichloromethane (DCM), ethyl acetate (EtOAc), and ethanol/water (EtOH/H_2_O) by ultrasound-assisted extraction (UAE). The extractions were performed in a 3L sonication reactor PEX 3 Sonifier (R.E.U.S, Contes, France), consisting of a stainless jug equipped with a double-layered mantle that allowed water to circulate using a cooling system to regulate the temperature. The base of the stainless reactor was equipped with a transducer operating at a frequency of 25 kHz, with a maximum output power from the generator of 150 W. Double extractions were performed for each type of solvent at room temperature. In total, 1 L of solvent was used for each extraction. Τhe extracts were then filtered and concentrated to dryness, and the dry residues were weighed. Table 1 summarizes the recovered extracts, their weight, and the respective extraction yields (% *w*/*w*).

### 2.4. Chromatographic Fractionation of Ethyl Acetate Extracts from Pseudobulbs and Roots

The ethyl acetate extracts of the pseudobulbs and roots were subjected to chromatographic separation to isolate their major secondary metabolites.

Initially, 3.7 g of the pseudobulbs’ ethyl acetate extract was fractionated in a vertical glass column (4 cm in width × 80 cm in length) packed with 300 g of 40–60 mesh silica gel. Silica slurry was prepared with c-Hex and was poured from the top of the column filling approximately 2/3 rd of the column, while the solvent was simultaneously drained to aid with the proper packing of the column. Sea sand (50–70 mesh particle size) was added on top of the slurry, to a height of 1 cm. The sample was placed on the top of the column, and the compounds were separated based on their polarity by passing different mixtures of c-Hex/EtOAc in increasing polarity order. In total, 422 fractions of 50 mL were collected and pooled based on their chemical similarity, resulting finally in 22 combined fractions (Ps-I to Ps-XXII). The weight and TLC chromatogram of all combined fractions are presented in Figure S6 (Supplementary Materials). The NMR and HRMS analysis of fraction Ps-IV revealed the presence of a mixture of two main compounds, i.e. dodecyl coumarate and dodecyl ferulate. The same spectroscopic analysis of Ps-V resulted in the identification of *β*-sitosterol as the main component of this fraction. Next, 95 mg of fraction Ps-VIII, was subjected to further purification analysis by 200–300 mesh silica gel CC. This step led to the isolation of two metabolites which were identified as 5-hydroxy-2-methoxy-1,4-phenanthrenquinone (3.9 mg) and ephemeranthoquinone B (1.87 mg). Fractions Ps-X and Ps-XI were combined, and a quantity of 122.5 mg was subjected to further separation by 200–300 mesh silica gel CC. This purification step resulted in the recovery of 28.5 mg of gigantol and 8.92 mg of orcinol in pure form. Finally, fraction Ps-XV was analyzed by preparative TLC, resulting in the recovery of two dibenzyls, which were identified as dihydroresveratrol (0.5 mg) and tristin (2.9 mg).

The ethyl acetate extract of the roots was subjected to a similar separation procedure. Initially, 3.6 g were fractionated by 40–60 mesh silica gel CC, using the same parameters as in the case of the pseudobulb extract. In total, 27 combined fractions were recovered (R-I to T-XXVII), their dry residue was weighed, and all fractions were analyzed by TLC (Figure S7, Supplementary Material). Fraction R-III (123.9 mg) was directly studied by NMR and HRMS, showing the presence of dodecyl coumarate as the main compound of the mixture. Fractions R-IV (114 mg) and R-V (259.8 mg) were also directly studied by NMR, showing the presence of *β*-sitosterol in high purity. Fractions R-IX and R-X were combined, and a quantity of 140 mg was separated by silica gel CC (200–300 mesh). This purification step resulted in the recovery of four pure compounds, which were identified as 3-hydroxy-2,4,7-trimethoxy-phenanthrene (3.3 mg), 5-hydroxy-2-methoxy-1,4-phenanthrenequinone (1.3 mg), ephemeranthroquinone B (14.5 mg), and marylaurencinol A (3.8 mg). Fraction R-XII was subjected to preparative TLC (20 mg), resulting in the isolation of (24R)-6*β*-hydroxy-24-ethyl-cholest-4-en-3-one (0.6 mg) and 3-hydroxy-2,4,7,8-tetramethoxy-phenanthrene (0.5 mg). The same separation method was followed for the purification of compounds of fraction R-XIV. Next, 20 mg of the fraction were analyzed by prep-TLC, leading to the recovery of 2.4 mg of bulbophyllanthrin and 1.6 mg of gigantol. Finally, further purification of fraction R-XIX by CC resulted in the recovery of 2.4 mg of cymbisamoquinone and 2.2 mg of a lignan identified as 9,9′-diacetyl-dehydrodiconiferyl alcohol. All CC purifications were performed using a 200–300 mesh silica gel stationary phase and mixtures of cyclohexane, EtOAc, and MeOH in increasing polarity order, as the elution solvents. The preparative TLC separations were performed on silica-gel glass plates (TLC silica gel 60 F254, Merck, Darmstadt, Germany) and developed with DCM/MeOH or c-Hex/EtOAc solvent mixtures.

### 2.5. TLC, HPLC, and NMR Analysis

TLC analyses of extracts, CC fractions and isolated compounds were performed on normal (silica gel 60 F254) and reversed phase (silica gel RP-18-F254S) TLC aluminum plates (Merck, Darmstadt, Germany). The absorption of the spots was studied in 254 and 366 nm, and in visible after spraying with sulfuric vanillin reagent (1:1 mixture of 5% (*v*/*v*) H_2_SO_4_ in MeOH and 10% (*w*/*v*) vanillin in MeOH), followed by heating at 120 °C for 1 min. Two different development systems for the TLCs were used; water/ACN 60:40 (*v*/*v*) for the reversed phase TLC development, while for the normal phase, a solvent system consisting of DCM/MeOH was used in various gradients. 

HPLC profiles of ethyl acetate extracts and isolated fractions of *Cymbidium* sp. were determined by HPLC-DAD. A Thermo Finnigan HPLC system (ON, Canada) equipped with a SpectraSystem P4000 pump, a SpectraSystem 1000 degasser, a SpectraSystem AS3000 automated injector, and a UV SpectraSystem UV6000LP diode-array detector was used under the control of the software platform ChromQuest 5.0 (Thermo Scientific, Bremen, Germany). The chromatographic column used for the extracts was a reversed-phase Supelco Analytical Discovery HS-C18 (250 × 4.6 mm, i.d. 5.0 μm). The analyses were performed at 25 °C, and the injection volume was 20 μL.

For the analysis of ethyl acetate extracts, a gradient method employing a binary mobile phase was used, where solvent (A) was water containing 0.1% formic acid (*v*/*v*) and solvent (B) was acetonitrile. The flow rate was set at 1.0 mL/min with a gradient elution starting at 20% (B), and the following chromatographic conditions were set: 0–3 min linear gradient to 30% (B); 3–11 min linear gradient to 35% (B); 11–25 min linear gradient to 50% (B); 25–37 min linear gradient to 100% (B); 37–40 min isocratic elution with 100% (B); 40–45 min linear gradient to 20% (B). For UV detection, the wavelengths employed were 280 and 350 nm.

Nuclear magnetic resonance (NMR) experiments (^1^H, ^13^C, COSY, NOESY, HSQC-DEPT, HMBC) were performed on a Bruker Avance III 600 spectrometer (Bruker Biospin GmbH, Rheinstetten, Germany). The isolated compounds were diluted in 600 μL of deuterated solvents (CDCl_3_ and MeOD), and the spectra were recorded at 305 K. The recovered spectra were calibrated at 7.26 (^1^H) and 77.0 ppm (^13^C) for CDCl_3_, and 3.31 (^1^H) and 49.15 ppm (^13^C) for CD_3_OD, respectively. The chemical shifts (*δ*) are expressed in ppm and the coupling constants (*J*) in Hz, while peaks are characterized as *s* (singlet), *brs* (broad singlet), *d* (doublet), *dd* (doublet of doublet), *t* (triplet), *q* (quartet), and *m* (multiplet).

### 2.6. UPLC-HRMS Analysis

UPLC-HRMS analyses were performed using an Acquity H-Class UPLC system (Waters Corp., Milford, USAhyphenated to a hybrid LTQ-Orbitrap Discovery XL (Thermo Scientific, Brehmen, Germany) mass spectrometer with an electrospray ionization (ESI) source. The chromatographic unit contained a quaternary pump, an autosampler, a vacuum degasser, and a temperature-controlled column. For the analyses, 200 μg of each sample were diluted with 1 mL of ACN/H_2_O 60:40 (*v*/*v*), and a volume of 10 μL was injected into the system. The chromatographic separation was performed on a Supelco Ascentis C18 (Sigma-Aldrich, Burlington, NJ, USA) column (150 × 2.1 mm, 3 μm) using, as the mobile phase, mixtures of water with 0.1% formic acid (solvent A) and acetonitrile (solvent B). The gradient elution method was as follows: 0 to 1 min, 5% (B); 1 to 15 min, from 5% (B) to 100% (B); 15 to 17 min, 100% (B); 17 to 17.5 min, from 100% (B) to 5% (B); and 17.5 to 20 min, 5% (B). The flow rate was set at 0.4 mL/min, while column temperature was kept stable at 40 °C.

The MS spectra were recorded in negative (ESI–) ionization modes, in the full scan mass range of *m*/*z* 115.0–1000.0, using a resolution of 30,000. The other parameters were as follows: the capillary temperature was 350 °C; the capillary and tube lens voltage was tuned to −30 and −100 V respectively; the source voltage was 2.70 kV (ESI–); the source current was 100 μA; the sheath gas and auxiliary gas flow rates were 40 and 10 arbitrary units, respectively. Xcalibur 2.0.7 (Thermo Scientific, Bremen, Germany) software was used for data acquisition and processing.

### 2.7. DPPH

The DPPH radical scavenging assay determines antioxidant activity using the stable free radical 2,2-diphenyl-1-picrylhydrazyl [24]. Initially, 190 μL of DPPH solution (314 μM, 12.4 mg dissolved in 100 mL of absolute ethanol) and 10 μL of the tested sample were dispensed into each well of 96-well microplate and were incubated for 30 min at room temperature, avoiding light exposure. The final tested concentrations of the extracts were 5 μg/mL, 10 μg/mL, 20 μg/mL, 50 μg/mL, 100 μg/mL, 250 μg/mL, and 500 μg/mL dissolved in DMSO. The reduction of the free radical is visible because of its color change from deep purple to yellow, and the absorbance was measured at 517 nm using a Tecan (Infinite 200 Pro series, Männedorf, Switzerland). The negative control contained 10 μL of DMSO, and Gallic Acid (IC_50_ = 100 μg/mL dissolved in DMSO) was used as the positive control due to its well-known antioxidant activity. Experiments were performed in triplicate.

The antioxidant activity was calculated by the following equation:

Inhibition (%) = (Abs A − Abs B)/(Abs A) × 100

where Abs A = Abs Control − Abs Control Blank and Abs B = Abs Sample − Abs Sample Blank. Abs Control is the absorbance of the negative control, and Abs Sample is the absorbance of the tested sample. Blanks consist of all the above reagents except the free radical reagent.

### 2.8. Tyrosinase Assay

Tyrosinase inhibitory activity was determined by the absorbance of dopachrome produced after the oxidation of L-dopamine (l-3, 4-dihydroxyphenylalanine, L-DOPA) using a previously described protocol with slight modifications [25]. Initially, 80 μL of PBS buffer (Na_2_HPO_4–_7H_2_O, NaH_2_PO_4_H_2_O, 0.067 M, pH = 6.8), 40 μL of the tested sample, and 40 μL of tyrosinase solution (92 U/mL in PBS buffer) were dispensed into each well of a 96-well microplate and were incubated for 10 min at room temperature, avoiding light exposure. Next, 40 μL of the substrate L-DOPA (2.5 mM dissolved in PBS buffer) was set onto the plate and was incubated for an additional 10 min at the same conditions.. The absorbance of the dopachrome was measured at 475 nm using a Tecan (Infinite 200 Pro series, Männedorf, Switzerland). The final tested concentrations of the extracts and pure compounds were 75 μg/mL, 150 μg/mL, 300 μg/mL, and 25 μΜ, 100 μΜ, 250 μΜ, respectively, dissolved in PBS buffer, and the percentage of DMSO was under 5% of the total volume inside the plate. The negative control of the assay was 5% DMSO PBS buffer, and the positive control was Kojic Acid (IC_50_ = 10 μM), a strong tyrosinase inhibitor. Experiments were performed in triplicate.

The tyrosinase inhibitory activity was calculated by the following equation:

Inhibition (%) = (Abs A − Abs B)/(Abs A) × 100

where Abs A = Abs Control − Abs Control Blank and Abs B = Abs Sample − Abs Sample Blank. Abs Control is the absorbance of PBS Buffer with 5% DMSO, tyrosinase enzyme, and substrate, and Abs Sample is the absorbance of the tested sample in the PBS buffer, tyrosinase enzyme, and substrate. Blanks consist of all the above reagents except the enzyme.

### 2.9. Elastase Assay

Elastase inhibitory activity was measured by the presence of *p*-nitroaniline, after the reaction of the elastase enzyme with the substrate N-succinyl-Ala-Ala-Ala-*p*-nitroanilide, using a previously described protocol with minor modifications [26]. At first, 70 μL of Trizma-base buffer (50 mM, pH = 7.5), 10 μL of the tested sample, and 5 μL of elastase solution (0.4725 U/mL dissolved in Trizma-base buffer) were mixed in the 96-well microplate and were incubated for 10 min at 37 °C, avoiding light exposure. Following this procedure, 15 μL of the substrate (2 mM dissolved in Trizma-base buffer) were added to every well of the plate, and then the plate was incubated for 30 min at 37 °C. The presence of *p*-nitroaniline was measured spectrophotometrically at 405 nm using a Tecan (Infinite 200 Pro series, Männedorf, Switzerland). The final tested concentrations of the extracts and pure compounds were 50 μg/mL, 100 μg/mL, 300 μg/mL, and 25 μΜ, 100 μΜ, 250 μΜ, respectively, dissolved in Trizma-base buffer, and the percentage of DMSO was under 5% of the total volume inside the plate. The negative control of the assay was 5% DMSO Trizma-base buffer, and the positive was Elastatinal (IC_100_ = 5 μg/mL and IC_50_ = 0.5 μg/mL), a strong irreversible competitive elastase inhibitor. Experiments were performed in triplicate.

The elastase inhibitory activity was calculated by the following equation:

Inhibition (%) = (Abs A − Abs B)/(Abs A) × 100

where Abs A = Abs Control − Abs Control Blank and Abs B = Abs Sample − Abs Sample Blank. Abs Control is the absorbance of Trizma-base Buffer with 5% DMSO, elastase enzyme, and substrate, and Abs Sample is the absorbance of the tested sample in the Trizma-base buffer, elastase enzyme, and substrate. Blanks consist of all the above reagents except the enzyme.

### 2.10. Collagenase Assay

Collagenase inhibitory activity was determined by a previously described enzymatic method with slight modifications, based on the fluorescence of the MMP-2 substrate after its degradation by collagenase [27]. In a 96-well black microplate, 50 μL of Tris-HCL buffer (10 mM, pH = 7.3), 25 μL of the tested sample (dissolved in Tris-HCL buffer), and 25 μL of collagenase solution (30 μg/mL dissolved in Tris-HCL buffer) were preincubated for 10 min at 37 °C, avoiding light exposure. Afterwards, 25 μL of MMP-2 substrate (50 μM dissolved in Tris-HCL buffer) were added, and the plate was incubated for an additional 30 min at 37 °C in the dark. The fluorescent intensity was measured at an excitation maximum of 320 nm and an emission maximum of 405 nm using a Tecan (Infinite 200 Pro series, Männedorf, Switzerland). Crude extracts were evaluated at 75 μg/mL, 150 μg/mL, and 300 μg/mL and pure compounds at 25 μΜ, 100 μΜ, and 250 μΜ, respectively, dissolved in PBS buffer, while the percentage of DMSO was under 5% of the total volume inside the plate. The negative control of the assay was 5% DMSO Tris-HCL buffer, and Phosphoramidon was the positive (IC_50_ = 6.9 μM), a strong metallo-endopeptidase inhibitor. Experiments were performed in triplicate. The collagenase inhibitory activity was calculated by the following equation:

Inhibition (%) = (Abs A − Abs B)/(Abs A) × 100

where Abs A = Abs Control − Abs Control Blank and Abs B = Abs Sample − Abs Sample Blank. Abs Control is the absorbance of Tris-HCL buffer with 5% DMSO, collagenase enzyme, and substrate, and Abs Sample is the absorbance of the tested sample in the Tris-HCL buffer, collagenase enzyme, and substrate. Blanks consist of all the above reagents except the enzyme.

### 2.11. Statistical Analysis

GraphPad Prism software 8.4.3 was used for all the analyses. The means are represented as mean ± SD (*n* = 3) for all the biological assays, and the occurrence of statistical differences among the data was evaluated by using one-way ANOVA only in the collagenase assay. All the samples were compared with the positive control and multiple comparisons of means were performed using Dunnett’s Test. Differences were considered significant at *p* < 0.05.

## 3. Results

### 3.1. In Vitro Biological Evaluation of Cymbidium sp. By-Products’ Extracts

#### 3.1.1. Antioxidant Potential of *Cymbidium* sp. Total Extracts

The DPPH reaction has been used extensively to compare and rank the antioxidant effectiveness of a wide range of natural extracts in thousands of studies [28]. It is considered a valid method for an initial quantitation of antioxidant (mostly phenol) content. In our case, the antioxidant activity of all initial extracts obtained from the flowers, leaves, pseudobulbs, and roots of *Cymbidium* sp. was determined on the basis of a DPPH free radical scavenging assay (Figure S1; Supplementary Materials). The IC_50_ value of each extract was calculated, and the results are presented in Table 2.

Among the different *Cymbidium* parts, the highest scavenging activity was presented by pseudobulbs and roots in all three different polarities, while leaves and flowers produced extracts with weak or no antioxidant activity. There are reports in the literature attempting to group the antioxidant activity of metabolites by IC_50_ values. Briefly, it has been proposed that samples with IC_50_ below 50 μg/mL have very strong antioxidant activities, while those with 50–100 μg/mL are strong; those with 101–150 μg/mL are moderate, and those with IC_50_ higher than 150 μg/mL are weak antioxidants [28]. Based on this classification, the ethyl acetate extracts of pseudobulbs and roots exhibited moderate antioxidant activity (IC_50_ = 114.18 μg/mL and 127.17 μg/mL, respectively) in contrast to the non-polar and polar extracts of the same plant parts, which presented weak activity (Table 2). Antioxidant capacity is the first factor in choosing the most suitable extract for dermo-cosmetic use. In our case, the ethyl acetate extracts of the pseudobulbs and roots are the most promising for further investigation.

#### 3.1.2. Determination of Anti-Tyrosinase, Anti-Elastase, and Anti-Collagenase Activity of Total Extracts

**Figure 2:**
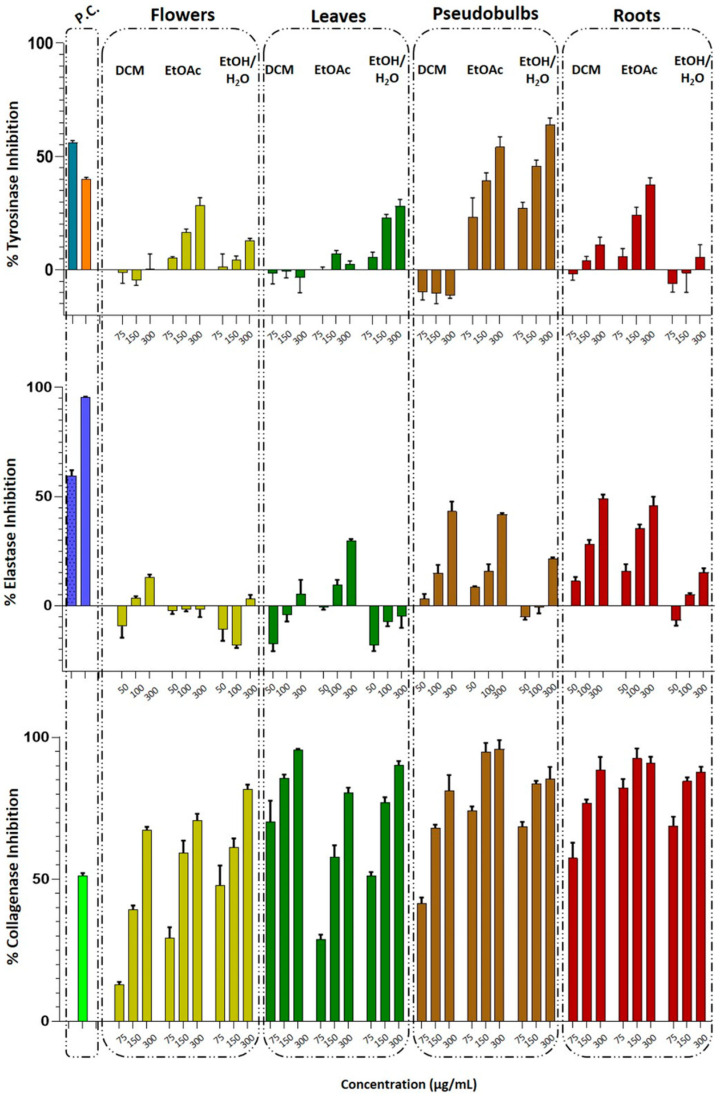
Tyrosinase, elastase, and collagenase inhibitory activity of extracts in three different concentrations. The yellow, green, brown, and red bars represent the flowers, leaves, pseudobulbs, and roots of the plant, respectively. Bars depict mean ± S.D., *n* = 3 independent experiments. P.C. = Positive Control. P.C. for Tyrosinase Assay is Kojic Acid (IC_50_ = 10 μM, left bar) and glycyrrhiza extract (IC_50_ = 1 μg/mL, right bar); for Elastase Assay, it is Elastatinal (IC_100_ = 5 μg/mL, right bar, IC_50_ = 0.5 μg/mL, left bar); for Collagenase, it is Phosphoramidon (IC_50_ = 6.9 μM).

Tyrosinase, elastase, and collagenase are involved in the pigmentation of the human skin and the processes of aging. Thus, the activity of these enzymes is used to test the efficiency of extracts and pure compounds for dermo-cosmetic applications [24,29]. Although there is now a plethora of studies regarding the activity of natural product extracts against elastase, tyrosinase, or collagenase enzymes, there are no reports regarding the effects of *Cymbidium* sp. by-products on these enzymes. This could be a benchmark for their use in the dermo-cosmetic industry, which would contribute to the so-called “circular economy” by reusing a by-product. With this concept in mind, all extracts obtained from the by-products of *Cymbidium* were evaluated for their inhibition properties on tyrosinase, elastase, and collagenase enzymes, and the results are presented in Figure 2.

For all the *Cymbidium* sp. by-product extracts, the enzymatic assay for tyrosinase enzyme was applied as previously described [24]. As the standard comparison for this enzymatic assay, the half minimum inhibitory concentration (IC_50_) of Kojic acid, a known tyrosinase inhibitor used in cosmetics for skin whitening, was used. The results of the assay (Figure 2) revealed that none of the DCM extracts of any plant parts was active, indicating that the DCM is not a suitable solvent for the extraction of antityrosinase active metabolites from *Cymbidium*. In contrast, ethyl acetate extracts, and particularly those obtained from flowers, pseudobulbs, and roots exhibited a dose-dependent inhibitory activity. More specifically, the pseudobulb ethyl acetate extract presented the higher antityrosinase activity, showing 26.70 ± 2.34%, 39.31± 2.84%, and 54.19 ± 3.72% inhibition at 75, 150, and 300 μg/mL, respectively, while root and flower ethyl acetate extracts were found to be less active, presenting 37.50 ± 2.54% and 28.29 ± 2.93% inhibition, respectively, at the higher concentration of 300 μg/mL. Regarding the hydroalcoholic extracts, only the pseudobulbs’ ethanol/water extract presented remarkable activity showing 27.26 ± 2.08%, 45.80 ± 2.15%, and 64.11 ± 2.34% inhibition at 75, 150, and 300 μg/mL, respectively.

Figure 2 summarizes the elastase and collagenase inhibitory activities of the twelve *Cymbidium* sp. by-product extracts. Considering elastase inhibition, only the less polar extracts of the pseudobulbs and roots exhibited moderate anti-elastase activity in a dose-dependent manner. More specifically, the DCM and ethyl acetate extracts of the pseudobulbs presented 44.23 ± 2.28% and 41.71 ± 0.61% inhibition at 300 μg/mL, respectively, while the same extracts obtained from roots showed 49.09 ± 1.50% and 45.99 ± 3.25% inhibition at the same concentration. All the other extracts, and especially those containing the polar constituents of the *Cymbidium* sp. by-products, were inactive. Regarding the collagenase inhibitory test, almost all the extracts presented important activity. In particular, eight of the twelve tested extracts (Table S8, Supplementary Material) presented statistically significant inhibitory activity even at the lower concentration of 75 μg/mL, compared with the positive control, as determined by one-way ANOVA (*p* < 0.001). Moreover, the most active extracts were found to be the ethyl acetate extracts of the pseudobulbs and roots, which showed high inhibitory activity (74.01 ± 1.32% and 82.79 ± 1.60% inhibition, respectively) at the concentration of 75 μg/mL (Figure 2).

Based on the above results, we can conclude that the ethyl acetate extracts of the pseudobulbs and roots showed the highest antioxidant capacity, as well as important inhibition activity against all three enzymes examined. Therefore, the above extracts were selected for further investigation in order to establish their chemical composition and to isolate and identify the active compounds.

### 3.2. Phytochemical Analysis of Pseudobulb and Root EtOAc Extracts

#### 3.2.1. Isolation of the Main Secondary Metabolites

The preliminary TLC and HPLC-UV/DAD analysis (Figures S2 and S3, Supplementary Material) of the ethyl acetate extracts of the pseudobulbs and roots presented a mixture of compounds with approximately 10 base peaks in each chromatogram. The comparison of respective chromatograms revealed some differences between the two extracts, especially regarding their main constituents. In order to isolate and biologically evaluate the main components of the active mixtures, both pseudobulb and root ethyl acetate extracts were submitted to further phytochemical analysis using silica gel column chromatography.

**Figure 3:**
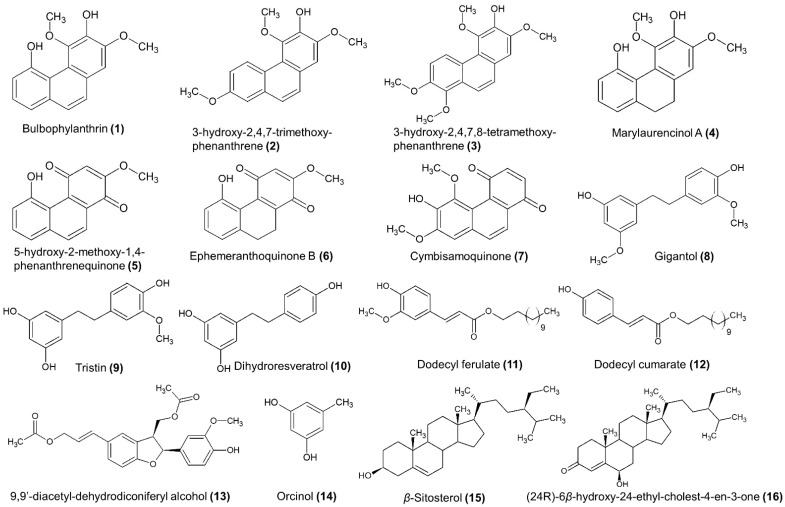
Structures of isolated compounds from pseudobulb and root ethyl acetate extracts.

Initially, the chromatographic separation was performed on the ethyl acetate extract of the roots. In total, 3.6 g were fractionated in a two-step separation procedure (see Section 2.4 at Materials and Methods), which finally resulted in the isolation of 12 secondary metabolites. Of these, four belong to phenanthrenes, three 1,4-phenanthrenequinones, one dibenzyl, one lignan, one phenolic acid derivative, and two sterols. More specifically, the analysis of the roots’ ethyl acetate extract led to the isolation of bulbophyllanthrin (**1**) [30], 3-hydroxy-2,4,7-trimethoxy-phenanthrene (**2**) [16], 3-hydroxy-2,4,7,8-tetramethoxy-phenanthrene (**3**) [31], marylaurencinol A (**4**) [18], 5-hydroxy-2-methoxy-1,4-phenanthrenequinone (**5**) [7], ephemeranthroquinone B (**6**) [18], cymbisamoquinone (**7**), gigantol (**8**) [32], dodecyl ferulate(**11**) [33], 9,9′-diacetyl-dihydrodiconiferyl alcohol (**13**) [34], *β*-sitosterol (**15**) [35], and (24R)-6*β*-hydroxy-24-ethyl-cholest-4-en-3-one (**16**) [36] (Figure 3). It is important to note that compound 7 (Figure 3), identified as 6-hydroxy-5,7-dimethoxy-1,4-phenanthrenequinone has not been described in the literature so far and thus it is considered a new natural product with the given name cymbisamoquinone.

The same chromatographic procedure was applied for the analysis of the ethyl acetate extract of the pseudobulbs. In total, 3.7 g of extract were analyzed, resulting in the isolation of eight secondary metabolites. Two 1,4-phenanthrenequinones, 5-hydroxy-2-methoxy-1,4-phenanthrenequinone (**5**) and ephemeranthroquinone B (**6**)), three dibenzyls (gigantol (**8**), tristin (**9**) [37], and dihydroresveratrol (**10**) [38]), one phenolic acid derivative (dodecyl coumarate (**12**) [39]), one simple phenolic compound (orcinol (**14**) [40]), and one sterol (*β*-sitosterol (**15**)).

The structure elucidation of the isolated compounds was achieved by NMR (1D and 2D) and HRMS analysis and verified by a comparison of the experimental data with those existing in literature. The ^1^H and ^13^C NMR data of all isolated compounds are presented in Tables S1–S7 (Supplementary Materials), while the 1D- and 2D-NMR spectra of the most important compounds are given on Figures S8–S17 (Supplementary Material).

In total, the phytochemical analysis of the two active extracts resulted in the isolation and structural elucidation of 16 secondary metabolites (Figure 3), of which four were phenanthrenes (**1**, **2**, **3**, and **4**), three were 1,4-phenanthrenequinones (**5**, **6**, and **7**), three were dibenzyls (**8**, **9**, and **10**), two were phenolic acid derivatives (**11** and **12**), one was a simple phenolic compound (**14**), one was a lignan (**13**), and two were sterols (**15** and **16**). Of these, eight metabolites were isolated solely from the roots’ ethyl acetate extract (**1**, **2**, **3**, **4**, **7**, **11**, **13**, and **16**) and four were isolated solely from the pseudobulbs’ ethyl acetate extract (**9**, **10**, **12**, and **14**), while four compounds were isolated from both extracts (**5**, **6**, **8**, and **15**). This fact shows that phenanthrenes are overexpressed mainly in the roots of *Cymbidium* sp., while the two isolated phenanthrenequinones, 5-hydroxy-2-methoxy-1,4-phenanthrenequinone (**5**) and ephemeranthroquinone B (**6**), as well the dibenzyl gigantol (**7**) and the sterol *β*-sitosterol, are major metabolites of both pseudobulbs and roots of *Cymbidium* sp.

It is important to note that phenanthrenes, 1,4-phenanthrenequinones, and dibenzyls are characteristic metabolites of the Orchidaceae family. Bulbophyllanthrin (**1**) has been previously isolated from *Dendrobium* sp. and *Bulbophyllum leopardium* with reference to antifibrotic activity in vitro [30], while marylaurencinol-A (**4**) was isolated from *Cymbidium* Great Flower “Marylaurencin” with antimicrobial activities [11]. Gigantol (**7**) and tristin (**8**) have mainly been referred to as isolated metabolites from *Dendrobium* sp., with gigantol displaying a wide range of bioactivities, including anti-oxidant, anti-mutagen, anti-platelet aggregation, anti-spasmodic and anti-ageing effects [41,42]. Furthermore, in the chemical category of 1,4-phenanthrenequinones, 5-hydroxy-2-methoxy-1,4-phenanthrenequinone (**5**) and ephemeranthoquinone-B (**6**) were isolated from *Cymbidium* sp., with 5-hydroxy-2-methoxy-1,4-phenanthrenequinone showing cytotoxic activity against human cell lung cancer (NCI-H187) cell line [21] and ephemeranthoquinone-B showing antimicrobial and antioxidant activities in vitro [18].

On the other hand, dihydroresveratrol (**10**), 9,9′-diacetyl-dihydrodiconiferyl alcohol (**13**), dodecyl ferulate (**11**), dodecyl coumarate (**12**), and (24R)-6*β*-hydroxy-24-ethyl-colest-4-en-3-one (**16**) have not been isolated again from other species of the *Orchidaceae* family. In the present work, these metabolites were isolated and identified from *Cymbidium* sp. for the first time. Finally, the isolation and structural characterization of the new natural product cymbisamoquinone (**7**) is described here for the first time.

Cymbisamoquinone (**7**) (6-hydroxy-5,7-dimethoxy-1,4-phenanthrenequinone) was isolated as a pure compound from the ethyl acetate extract of the roots of *Cymbidium* sp. The structure elucidation was based on the 1D and 2D NMR and LC-HRMS analysis, as well as on the comparison with the spectroscopic data of the similar compound 7-hydroxy-5,6-dimethoxy-1,4-phenanthrenequinone, which was isolated from the ethyl acetate extract of *Dendrobium moniliforme* [43]. The HRMS spectrum of cymbisamoquinone presented a pseudomolecular ion at *m*/*z* 283.0615 [M-H]^−^ (suggested EC C_16_H_11_O_5_ and RDBeq. 11). The proton NMR spectrum of 6-hydroxy-5,7-dimethoxy-1,4-phenanthrenequinone (in CDCl_3_) presents two double peaks with *J* = 8.4 Hz at 7.95 and 7.89 ppm (H-10 and H9, respectively), two double peaks (*J* = 10.2 Hz) at 7.07 and 6.84 ppm (H-3 and H-2, respectively), one singlet at 6.99 ppm (H-8), one broad singlet at 6.22 ppm (6-OH) and two single peaks at 4.07 and 3.93 ppm, corresponding to methoxy protons 7-OCH_3_ and 5-OCH_3,_ respectively (Table S2 and Figure S12a, Supplementary Material). Both ^1^H and ^13^C NMR spectroscopic data of cymbisamoquinone are very similar to the corresponding data of 7-hydroxy-5,6-dimethoxy-1,4-phenanthrenequinone [43]. The main differences between the spectra of the two isomers are observed in the signals H-8 and C-8, as well as in the corresponding signals of the two methoxy groups, thus suggesting a different arrangement of these groups in positions 5, 6, and 7 of the 1,4-phenanthrenequinone skeleton. The study of the HMBC spectrum confirmed the presence of the methoxy groups at positions 5 and 7 (Figure S12d, Supplementary Material). The *J^3^* coupling signals between the peaks at *δ* 6.21 (-OH) and *δ* 4.07 (7-OCH_3_) with the carbon resonating at 150.5 ppm, as well as the *J^3^* signals between the peaks at *δ* 6.21 (-OH) and *δ* 3.93 (5-OCH_3_) with the carbon at 142.0 ppm, place the free hydroxyl group at position 6 and between the two methoxy groups. Moreover, the *J^3^* coupling signal of the peak at *δ* 7.89 (H-9) with the carbon at 102.3 ppm verifies the presence of the aromatic proton at position 8. Further study of 2D-NMR spectra (COSY, HSQC-DEPT, HMBC, Figure S12b–d, Supplementary Material) verified the structure of 6-hydroxy-5,7-dimethoxy-1,4-phenanthrenequinone.

#### 3.2.2. UPLC-HRMS/MS Analysis of Pseudobulb and Root Ethyl Acetate Extracts

The active ethyl acetate extracts of pseudobulbs and roots were further analyzed by UPLC-HRMS in negative mode in order to determine in depth their chemical composition. Orbitrap analyzer has the ability to provide accurate mass measurements (Δm < 2–3 ppm) and enabled the identification of compounds with high confidence. The identification process was based on HRMS spectra, suggested Elemental Composition (EC), RDBeq values, and literature data. In addition, the chemical structures of the isolated compounds were verified by analyzing them as reference standards. The BP chromatograms of both extracts revealed a rich content of compounds, especially between 6 and 14 min (Figures S4 and S5, Supplementary Material). The following analysis led to the tentative or unambiguous (comparison with reference compounds) identification of 48 metabolites which belong to seven chemical categories, i.e., simple phenols and phenolic acids, dibenzyls/stilbenes, phenanthrenes/phenanthrenequinones, flavonoids, and alkaloids. Table 3 summarizes the identified compounds, their Rt, pseudomolecular ions (*m*/*z*), EC, and RDBeq value, as well as their presence in the two analyzed extracts.

Thirteen of the above detected metabolites (in bold), were unambiguously identified by comparing their R*t* and ECs with those of the isolated compounds (Section 3.2.1). The other metabolites of Table 3 were tentatively identified by comparing the accurate masses observed in the chromatograms with those provided in the literature, considering also the polarity and elution order of the compounds. Phenanthrenes and 1,4-phenanthrenequinones (22–35, Table 3) were found to be the largest categories of compounds in the analyzed ethyl acetate extracts. Of these, phenanthrenes are expressed mainly in the roots, while its presence in the pseudobulbs’ extract was found only in traces. In contrast, the metabolites belonging to 1,4-phenanthrenequinones (23, 25, 26 and 33, Table 3) are present in both pseudobulb and root extracts. Moreover, six dibenzyls (13–18) and three stilbenes (19–21, Table 3) were detected in both extracts, with gigantol (*m*/*z* 273.1135) and tristin (*m*/*z* 259.0976) found to be the most abundant.

The above analysis resulted additionally in the identification of six flavonoids (36–41, Table 3), which were found mainly in the pseudobulbs’ extract, nine simple phenols and phenolic acids (1–9), two phenolic acid esters of fatty alcohols (11–12, Table 3), one lignan (10, Table 3), three alkaloids (45–47, Table 3), three organic acids (42–44, Table 3), and one triterpene (48, Table 3). It is important to note that orcinol (*m*/*z* 123.0453), which was isolated as a main compound from the ethyl acetate extract of pseudobulbs, was absent from the ethyl acetate extract of roots, while 9,9′-diacetyl-dehydrodiconiferyl alcohol (*m*/*z* 441.2648) isolated from the root ethyl acetate extract was found only in traces in the corresponding pseudobulb extract.

It is important to note that both analyzed extracts have been recovered using the same extraction procedure and in approximately the same yield (Table 1) while their antioxidant (Table 2) and inhibitory activity against tyrosinase, elastase, and collagenase were also similar (Figure 2). Nevertheless, the above UPLC-HRMS analysis showed a significant differentiation of the two extracts regarding their chemical composition. Taking into account the fact that the differentiation in chemical composition directly affects the safety of a mixture, the results of the above analysis could contribute significantly to the selection of the appropriate extract for further exploitation as a dermo-cosmetic agent.

#### 3.2.3. Determination of Anti-Tyrosinase, Anti-Elastase, and Anti-Collagenase Activity of Pure Compounds

**Figure 4:**
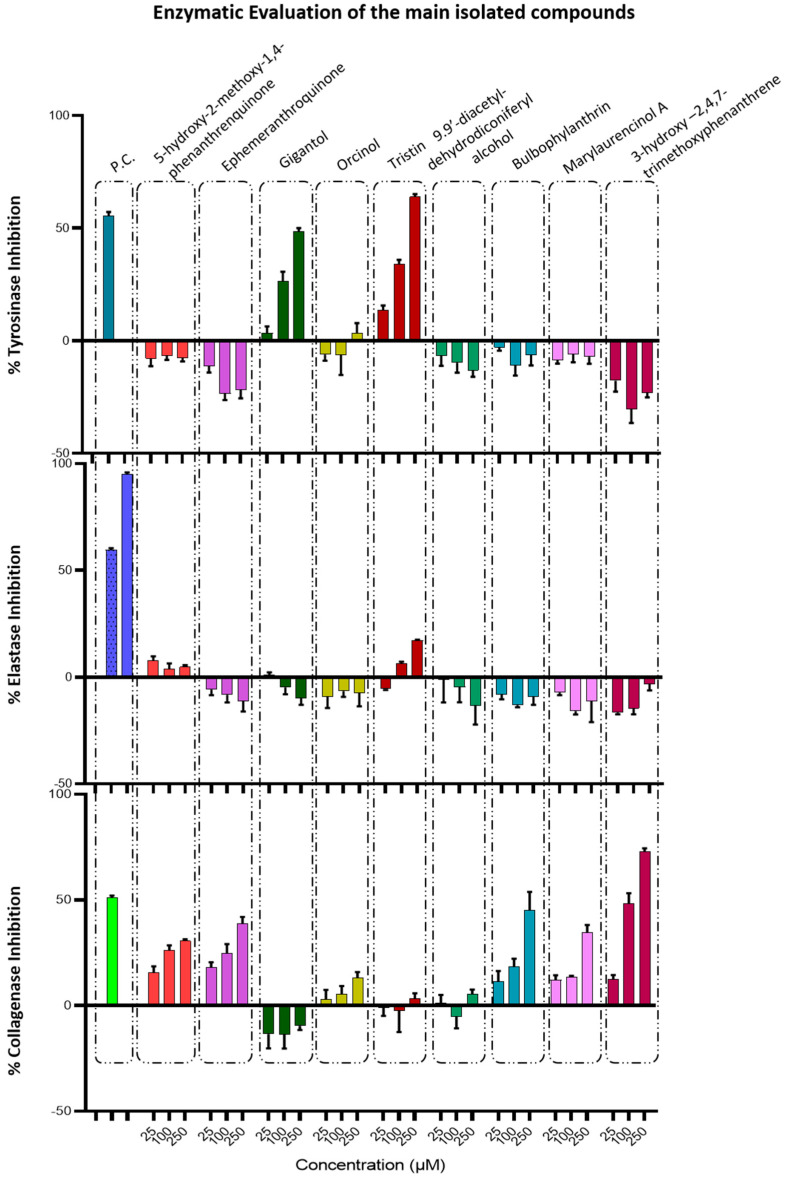
Tyrosinase, Elastase, and Collagenase inhibitory activity of isolated compounds. Bars depict mean ± S.D., *n* = 3 independent experiments. P.C. = Positive Control. P.C. for tyrosinase assay is Kojic Acid (IC_50_ = 10 μM); for elastase assay, it is Elastatinal (IC_100_ = 5 μg/mL, IC_50_ = 0.5 μg/mL); for collagenase, it is Phosphoramidon (IC_50_ = 6.9 μM).

In order to investigate whether the action on the aforementioned enzymes is due to total extracts or isolated compounds, we proceeded with the determination of the anti-tyrosinase, anti-elastase, and anti-collagenase activity of nine selected pure compounds obtained from the separation analysis of pseudobulb and root ethyl acetate extracts. The tested compounds were bulbophyllanthrin, 3-hydroxy-2,4,7-trimethoxy-phenanthrene, marylaurencinol A, 5-hydroxy-2-methoxy-1,4-phenanthrenequinone, ephemeranthroquinone B, gigantol, tristin, orcinol, and 9,9′-diacetyl-dehydrodiconiferyl alcohol. These compounds have been recognized as the major phenolic compounds of the active pseudobulb and root ethyl acetate extracts and were recovered in pure form and in sufficient amounts during the chromatographic isolation procedures. Moreover, three of the above tested compounds (5-hydroxy-2-methoxy-1,4-phenanthrenequinone, ephemeranthroquinone B, and gigantol) are present in both active extracts; thus, the detection of their activity against all three enzymes would help to better interpret the activity of the total extracts. The inhibitory activity of the tested compounds against the three enzymes are presented in Figure 4.

From all the tested compounds, only the dibenzyls, gigantol, and tristin presented a moderate, dose-dependent, inhibition activity on tyrosinase (Figure 4). Gigantol is a dibenzyl phenol, which is also isolated from *Cymbidium* sp. with one methoxy moiety on each aromatic ring. It is known that hydroquinone, which can derive from the metabolism of dibenzyl type derivatives (dihydroxybenzene), seems mainly to affect melanocytes with active tyrosinase activity [48]. Furthermore, hydroxystilbene compounds such as resveratrol, which are naturally contained in many plant extracts, have potent inhibitory effects on mushroom tyrosinase. Resveratrol is probably metabolized by tyrosinase, leading to the formation of an *o*-hydroxy derivative, which is a competitive inhibitor of the enzyme [48]. It is possible that the metabolites of gigantol may be responsible for the activity of this compound towards tyrosinase activity; tristin belongs to same chemical category of dibenzyls, which differ from gigantol due to the presence of a free hydroxyl group in position 3′; this may alter its activity towards the enzyme active site. Both gigantol and tristin were found to be non-active against elastase and collagenase enzymes. Although the research for isolated compounds that could inhibit tyrosinase, elastase, and collagenase has been extensive, there are no reports regarding the effects of gigantol or tristin on these enzymes.

Phenanthrenes and 1,4-phenanthenequinones presented moderate dose-dependent, inhibition activity on the collagenase enzyme, while there was no activity against tyrosinase and elastase (Figure 4). Of these, 3-hydroxy-2,4,7-trimethoxy-phenanthrene was found to be the most active, presenting 12.62 ± 1.48%, 50.05 ± 2.67%, and 72.73 ± 1.38% inhibition at 25, 100, and 250 μM, respectively. Finally, orcinol and 9,9′-diacetyl-dehydrodiconiferyl alcohol were inactive against all three tested enzymes.

It is important to note that all the above tested compounds were inactive on the elastase enzyme, even if the initial ethyl acetate extracts were found to have moderate anti-elastase activity. This activity of the total extracts is probably connected to the minor compounds, which are degraded when we focus only on the main components. On the other hand, the major phenanthrenes and anthraquinones are capable to express anti-collagenase activity, but they have no impact on the tyrosinase enzyme. The opposite happens with the other main class of dibenzyls, which present only anti-tyrosinase activity. In contrast to the specific activity of the isolated compounds, the total ethyl acetate extracts are able to sufficiently inhibit all three enzymes. The above experimental findings can be a guide to the better exploitation of *Cymbidium* sp. by-products.

## 4. Conclusions

Since melanin, elastin, and collagen maintain skin health in terms of integrity and elasticity, over-expression of the corresponding enzymes contributes to hyperpigmentation, undesired wrinkles, and skin aging. Our findings, presented herein, highlight the inclusion of *Cymbidium* sp. by-products (pseudobulbs and roots) as a potential anti-ageing and rejuvenator ingredient in many dermo-cosmetic formulations. The phytochemical analysis of these promising extracts resulted in the identification of 48 metabolites and the isolation of sixteen major compounds, of which one was found to be a new natural product. The biological evaluation of the isolated compounds linked specific substances to the inhibition of specific enzymes. However, from our evaluation of the activity of selected *Cymbidium* sp. extracts against tyrosinase, elastase, and collagenase, along with isolated compounds, we can conclude that the use of the total extract as a substitute product for dermo-cosmetic use is more advantageous than the time-consuming and costly process of isolating specific metabolites. Therefore, the process of extracting a by-product of the cultivated orchid to produce a value-added product for dermo-cosmetic use could be an example of the so-called “circular economy” in the Greek islands of North Aegean Sea.

## Figures and Tables

**Table 1:** Extract weight (g) and their % (*w*/*w*) yields from 400 g of powdered shade-dried flowers, leaves, pseudobulbs, and roots of *Cymbidium* sp.

Part of the Plant	Extraction Solvent	Extract Weight (g)	Yield (% *w*/*w*)
Flowers	DCM	6.97	1.74
	EtOAc	6.46	1.61
	EtOH/H_2_O	11.09	2.77
Leaves	DCM	8.36	2.09
	EtOAc	7.81	1.95
	EtOH/H_2_O	11.3	2.82
Pseudobulbs	DCM	7.15	1.79
	EtOAc	3.69	0.92
	EtOH/H_2_O	14.83	3.70
Roots	DCM	2.06	0.65
	EtOAc	3.68	0.92
	EtOH/H_2_O	9.54	2.39

**Table 2:** IC_50_ values of DPPH radical scavenging activity of DCM, EtOAc, and EtOH/H_2_O extracts from the different parts of *Cymbidium* sp. The non-active extracts are marked as NA.

Part of Plant	Extraction Solvent	IC_50_ (μg/mL)
Flowers (Fl)	DCM	NA
	EtOAc	NA
	EtOH/H_2_O	NA
Leaves (L)	DCM	NA
	EtOAc	NA
	EtOH/H_2_O	NA
Pseudobulbs (Ps)	DCM	165.78
	EtOAc	114.18
	EtOH/H_2_O	233.32
Roots (R)	DCM	174.61
	EtOAc	127.17
	EtOH/H_2_O	304.02

**Table 3:** Secondary metabolites identified in ethyl acetate extracts of pseudobulbs (Ps) and roots (R) of *Cymbidium* sp. The numbers in bold are referred to metabolites which have been isolated from the analyzed extracts.

No.	R*t* (min)	Experimental [M-H]^−^	Suggested EC	Delta (ppm)	RDB Eq.	Compound	EtOAc Extract		Ref.
							Ps	R	
	Phenols, Phenolic acids, and derivatives								
1	3.74	123.0453	C_7_H_7_O_2_	1.44	4.5	Orcinol (**14**)	+	-	*
2	4.12	137.0245	C_7_H_5_O_3_	0.97	5.5	Salicylic acid	+	+	[1]
3	4.14	137.0245	C_7_H_5_O_3_	0.9	5.5	*p*-hydroxybenzoic acid	+	+	[1]
4	4.17	353.0883	C_16_H_17_O_9_	1.46	8.5	Chlorogenic acid	tr	tr	[24]
5	5.18	193.0508	C_10_H_9_O_4_	0.77	6.5	Ferulic acid	-	tr	[1]
6	5.37	163.0401	C_9_H_7_O_3_	0.75	6.5	*p*-coumaric acid	tr	tr	[1]
7	5.55	151.0403	C_8_H_7_O_3_	1.54	5.5	Vanillin	tr	tr	[29]
8	5.58	167.0352	C_8_H_7_O_4_	1.49	5.5	Vanillic acid	tr	tr	[44]
9	7.85	153.0152	C_7_H_5_O_4_	1.23	5.5	Protocatechuic acid	+	+	[44]
10	13.9	441.2648	C_27_H_37_O_5_	0.821	9.5	9,9′-diacetyl-dehydrodiconiferyl alcohol (**13**)	tr	+	*
11	14.06	361.2388	C_22_H_33_O_4_	1.02	6.5	Dodecyl ferulate (**11**)	+	+	*
12	14.86	331.2283	C_21_H_31_O_3_	1.21	6.5	Dodecyl coumarate (**12**)	+	+	*
	Dibenzyls and Stilbens								
13	6.99	259.0976	C_15_H_15_O_4_	0.07	8.5	Tristin (**9**)	**+**	+	*
14	7.11	229.0872	C_14_H_13_O_3_	0.67	8.5	Dihydroresveratrol (**10**)	**+**	tr	*
15	7.4	259.0976	C_15_H_15_O_4_	0.3	8.5	Dendrosinen C	+	+	[36]
16	8.66	273.1135	C_16_H_17_O_4_	1.09	8.5	Gigantol (**8**)	**+**	+	*
17	8.7	289.1082	C_16_H_17_O_5_	0.357	8.5	Dendrosinen A	+	+	[36]
18	8.72	243.1028	C_15_H_15_O_3_	0.67	8.5	Batatasin III	+	+	[12]
19	8.85	227.0714	C_14_H_11_O_3_	0.54	9.5	Resveratrol	+	+	[12]
20	9.77	301.1083	C_17_H_17_O_5_	0.11	9.5	Phoyunbene A or Phoyunbene B	+	+	[45]
21	10.0	241.0871	C_15_H_13_O_3_	−0.24	9.5	Thunalbene, Pholidotol D, Lusianthridin	tr	tr	[12]
	Phenanthrenes and 1,4-phenanthrenquinones								
22	5.85	243.0662	C_14_H_11_O_4_	0.115	9.5	Tetrahydroxy-9,10-dehydrophenanthrene	tr	+	
23	7.43	283.0615	C_16_H_11_O_5_	0.93	11.5	Denbinobin, Isoxoflaccidin	+	+	[46]
24	7.58	287.0925	C_16_H_15_O_5_	1.26	9.5	Coeloginanthridin, or 1,3,5-trihydroxy-2,4-dimethoxy-9,10-dihydrophenanthrene	tr	+	[2]
25	7.77	253.0508	C_15_H_9_O_4_	1.06	11.5	5-hydroxy-2-methoxy-1,4-phenanthrenequinone (**5**)	+	+	*
26	8.16	283.0615	C_16_H_11_O_5_	0.93	11.5	Cymbisamoquinone (**7**)	+	+	*
27	8.39	257.0817	C_15_H_13_O_4_	−0.90	9.5	Trihydroxy-methoxy-9,10-dihydrophenanthrene	tr	+	[1]
28	9.55	283.0977	C_17_H_15_O_4_	0.38	10.5	3-hydroxy-2,4,7-trimethoxy-phenanthrene (**2**)	tr	+	*
29	9.89	269.0821	C_16_H_13_O_4_	0.81	10.5	Bulbophyllanthrin (**1**)	tr	+	*
30	10.01	271.0987	C_16_H_15_O_4_	0.84	9.5	Marylaurencinol A (**4**)	tr	+	*
31	10.04	241.0871	C_15_H_13_O_3_	0.25	9.5	Hircinol or Coelonin	tr	+	[7]
32	10.06	479.1506	C_30_H_23_O_6_	1.228	19.5	Blestriarene B	+	+	[2]
33	10.4	255.0665	C_15_H_11_O_4_	0.62	10.5	Ephemeranthroquinone B (**6**)	+	+	*
34	10.45	313.1084	C_17_H_15_O_4_	0.71	10.5	3-hydroxy-2,4,7,8-tetramethoxy-phenanthrene (**3**)	tr	+	*
35	11.41	461.1608	C_27_H_25_O_7_	0.875	15.5	Pleionesins C	+	+	[47]
	Flavonoids								
36	7.10	285.0407	C_15_H_9_O_6_	0.87	11.5	Kaempherol	+	tr	[2]
37	7.10	285.0407	C_15_H_9_O_6_	0.87	11.5	Luteolin	+	tr	[29]
38	7.18	301.0357	C_15_H_9_O_7_	0.88	11.5	Quercetin	+	tr	[2]
39	7.29	269.0456	C_15_H_9_O_5_	0.38	11.5	Apigenin	+	tr	[26]
40	7.39	299.0563	C_16_H_11_O_6_	0.76	11.5	Diosmetin	+	tr	[29]
41	8.03	303.0513	C_15_H_11_O_7_	1.49	10.5	Taxifolin	+	tr	[29]
	Other compounds								
42	0.79	195.0512	C_6_H_11_O_7_	0.9	1.5	Gluconic acid or galactonic acid	+	+	[29]
43	0.88	133.0143	C_4_H_5_O_5_	0.85	2.5	Malic acid	+	+	[29]
44	1.10	191.0199	C_6_H_7_O_7_	1.28	3.5	Citric acid	tr	tr	[29]
45	6.67	312.1243	C_18_H_18_O_4_N	0.636	10.5	Moupinamide	+	+	[1]
46	6.67	282.1138	C_17_H_16_O_3_N	1.04	10.5	Paprazine	+	+	[1]
47	7.03	342.1349	C_19_H_20_O_5_N	0.655	10.5	n-trans-feruloyl 4′ *O* methyldopamine	+	+	[1]
48	15.18	455.3532	C_30_H_47_O_3_	0.44	7.5	Oleanolic acid	+	tr	[29]

* Isolated compounds as reference standards.

## Data Availability

Data is contained within the article or supplementary material.

## Supplementary Materials

The following are available online at https://www.mdpi.com/article/10.3390/antiox11010101/s1, Table S1: NMR data of (**1**), (**2**), (**3**), and (**4**) in CDCl_3_ (600 MHz, δ ppm), Table S2: NMR data of (**5**), (**6**), and (**7**) in CDCl_3_ (600 MHz, δ ppm), Table S3: NMR data of (**8**), (**9**), and (**10**) in CDCl_3_ (600 MHz, δ ppm), Table S4: NMR data of (**11**) and (**12**) in CDCl_3_ (600 MHz, δ ppm), Table S5: NMR data of (**13**) in CDCl_3_ (600 MHz, δ ppm), Table S6: NMR data of (**14**) in CDCl_3_ (600 MHz, δ ppm), Table S7: NMR data of (**15**) and (**16**) in CDCl_3_ (600 MHz, δ ppm), Table S8: P-values after one-way Anova on collagenase assay, Figure S1: DPPH radical scavenging activity of *Cymbidium* sp. extracts, Figure S2: TLC profiles of *Cymbidium* sp. bulb and root EtOAc extracts, Figure S3: HPLC-UV/DAD chromatogram of *Cymbidium* sp. bulbs and roots at 280nm, Figure S4: TIC chromatogram of EtOAc total extract from *Cymbidium* sp pseudobulbs, Figure S5: TIC chromatogram of EtOAc total extract *Cymbidium* sp. roots, Figure S6: TLC chromatogram and weights of combined fractions obtained from the EtOAc pseudobulb extract through silica gel CC., Figure S7: TLC chromatogram and weights of combined fractions obtained from the EtOAc root extract through silica gel CC., Figure S8: NMR spectra of compound 1 recorded in CDCl3 at 600 MHz; (a) 1H-NMR, (b) COSY, (c) HSQC-DEPT, (d) HMBC, Figure S9: NMR spectra of (**4**) recorded in CDCl_3_ at 600 MHz; (a) ^1^H-NMR, (b) COSY, (c) HSQC-DEPT, (d) HMBC, Figure S10: NMR spectra of (**5**) recorded in CDCl_3_ at 600 MHz; (a) ^1^H-NMR, (b) COSY, (c) HSQC-DEPT, d) HMBC, Figure S11: ^1^H-NMR spectrum of (**6**) recorded in CDCl_3_ at 600 MHz, Figure S12: NMR spectra of (**7**) recorded in CDCl_3_ at 600 MHz; (a) ^1^H-NMR, (b) COSY, c) HSQC-DEPT, d) HMBC, Figure S13: NMR spectra of (**8**) recorded in CDCl_3_ at 600 MHz; (a) ^1^H-NMR, (b) COSY, (c) HSQC-DEPT, (d) HMBC, Figure S14: NMR spectra of (**11**) recorded in CDCl_3_ at 600 MHz; (a) ^1^H-NMR, (b) COSY, (c) HSQC-DEPT, (d) HMBC, Figure S15: NMR spectra of (**13**) recorded in CDCl_3_ at 600 MHz; (a) ^1^H-NMR, (b) COSY, (c) HSQC-DEPT, (d) HMBC, Figure S16: NMR spectra of (**14**) recorded in CDCl_3_ at 600 MHz; (a) ^1^H-NMR, (b) COSY, (c) HSQC-DEPT, (d) HMBCR. Figure S17: NMR spectra of (**16**) recorded in CDCl_3_ at 600 MHz; (a) ^1^H-NMR, (b) COSY, (c) HSQC-DEPT, (d) HMBC.
