# Supplementary material for: Phytochemical Analysis and Dermo-Cosmetic Evaluation of Cymbidium sp. (Orchidaceae) Cultivation By-Products
Source: Antioxidants (Basel). 2021 Dec 31;11(1):101. doi: 10.3390/antiox11010101 (PMC8772768; doi:10.3390/antiox11010101)
Supplement: Supplementary file 1 [file antioxidants-11-00101-s001.zip › antioxidants-1522091-supplementary.pdf]

Supplementary material

Phytochemical Analysis and Dermo-Cosmetic Evaluation of *Cymbidium* sp. (Orchidaceae) cultivation by-products

Evangelos Axiotis[1,2], Apostolis Angelis[1*], Lemonia Antoniadi[1], Eleftherios A. Petrakis[1] and Leandros A. Skaltsounis[1,2*]

[1]*Division of Pharmacognosy and Natural Products Chemistry, Department of Pharmacy, National and Kapodistrian University of Athens*

[2]*Natural Products Research Center "NatProAegean", Gera, Lesvos, Greece.*

Corresponding authors: Apostolis Angelis

Leandros A. Skaltsounis

E-mail address: aangjel@pharm.uoa.gr ; skaltsounis@pharm.uoa.gr

Contents

Table S1: NMR data of compounds 1, 2, 3, and 4 in $CDCl_3$ (600 MHz, δ ppm).

Table S2: NMR data of compounds 5, 6 and 7 in $CDCl_3$ (600 MHz, δ ppm).

Table S3: NMR data of compounds 8, 9, and 10 in $CDCl_3$ (600 MHz, δ ppm).

Table S4: NMR data of compounds 11 and 12 in $CDCl_3$ (600 MHz, δ ppm).

Table S5: NMR data of compound 13 in $CDCl_3$ (600 MHz, δ ppm).

Table S6: NMR data of compound 14 in $CDCl_3$ (600 MHz, δ ppm).

Table S7: NMR data of compounds 15 and 16 in $CDCl_3$ (600 MHz, δ ppm).

Table S8: P-values after one-way Anova by Dunnett's multiple comparisons test against the positive control.

Figure S1: DPPH radical scavenging activity of *Cymbidium* sp. extracts from different plant parts.

Figure S2: TLC chromatograms of EtOAc pseudobulb and root extracts from *Cymbidium* sp.

Figure S3: HPLC-DAD chromatogram of EtOAc total extracts from *Cymbidium* sp. bulbs and roots at 280 nm.

Figure S4: BP–UPLC-HRMS chromatogram of EtOAc total extract from *Cymbidium* sp. pseudobulbs.

Figure S5: BP–UPLC-HRMS chromatogram of EtOAc total extract from *Cymbidium* sp. roots.

Figure S6: TLC chromatogram and weights of combined fractions obtained from the EtOAc pseudobulb extract through silica gel CC.

Figure S7: TLC chromatogram and weights of combined fractions obtained from the EtOAc root extract through silica gel CC.

Figure S8: NMR spectra of **compound 1** recorded in $CDCl_3$ at 600 MHz; (a) ^{1}H-NMR, (b) COSY, (c) HSQC-DEPT, (d) HMBC.

Figure S9: NMR spectra of **compound 4** recorded in $CDCl_3$ at 600 MHz; (a) ^{1}H-NMR, (b) COSY, (c) HSQC-DEPT, (d) HMBC.

Figure S10: NMR spectra of **compound 5** recorded in $CDCl_3$ at 600 MHz; (a) ^{1}H-NMR, (b) COSY, (c) HSQC-DEPT, d) HMBC.

Figure S11: ^{1}H-NMR spectrum of **compound 6** recorded in $CDCl_3$ at 600 MHz.

Figure S12: NMR spectra of **compound 7** recorded in $CDCl_3$ at 600 MHz; (a) ^{1}H-NMR, (b) COSY, c) HSQC-DEPT, d) HMBC.

Figure S13: NMR spectra of **compound 8** recorded in $CDCl_3$ at 600 MHz; (a) ^{1}H-NMR, (b) COSY, (c) HSQC-DEPT, (d) HMBC.

Figure S14: NMR spectra of **compound 11** recorded in $CDCl_3$ at 600 MHz; (a) ^{1}H-NMR, (b) COSY, (c) HSQC-DEPT, (d) HMBC.

Figure S15: NMR spectra of **compound 13** recorded in $CDCl_3$ at 600 MHz; (a) ^{1}H-NMR, (b) COSY, (c) HSQC-DEPT, (d) HMBC.

Figure S16: NMR spectra of **compound 14** recorded in $CDCl_3$ at 600 MHz; (a) ^{1}H-NMR, (b) COSY, (c) HSQC-DEPT, (d) HMBC.

Figure S17: NMR spectra of **compound 16** recorded in $CDCl_3$ at 600 MHz; (a) ^{1}H-NMR, (b) COSY, (c) HSQC-DEPT, (d) HMBC.

Table S1: NMR data of compounds **1**, **2**, **3**, and **4** in $CDCl_3$ (600 MHz, δ ppm).

	**Compound 1**		**Compound 2**		**Compound 3**		**Compound 4**	
	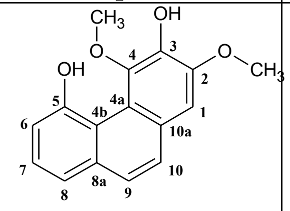		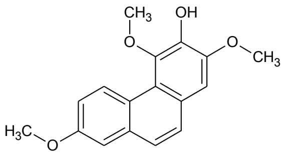		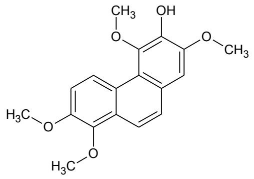		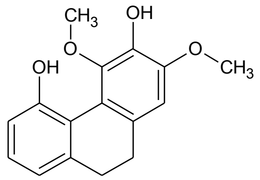	
	**[1]H (*J* in Hz)**	**[13]C**	**[1]H (*J* in Hz)**	**[13]C**	**[1]H (*J* in Hz)**	**[13]C**	**[1]H (*J* in Hz)**	**[13]C**
1	7.17 s	106.0	7.08 s	104.2	7.03 s	104.7	6.71 s	107.8
2		146.9		146.1		146.7		145.6
3		138.9		138.7		138.9		136.6
4		140.8		143.5		144.0		141.9
4a		117.1		118.6		118.6		118.9
4b		117.7		123.0		124.8		117.3
5		154.3	9.33 d (9.3)	128.1	9.13 d (9.5)	117.8		153.0
6	7.22 dd (7.8/1.4)	115.7	7.25 m	116.6	7.28 d (9.5)	116.7	6.96 dd (7.8/1.2)	118.0
7	7.49 t (7.8)	127.1		156.9		148.7	7.16 t (7.8)	128.3
8	7.41 dd (7.8/1.4)	120.3	7.23 m	108.2		143.1	6.85 dd (7.8/1.2)	119.7
8a		134.6		132.9		127.6	2.72 m (2H)	140.4
9	7.58 d (8.8)	126.9	7.53 d (8.9)	125.1	7.93 d (9.3)	125.5	2.66 m (2H)	31.0
10	7.50 d (8.8)	126.1	7.58 d (8.9)	127.0	7.54 d (9.3)	126.8		30.6
10a		127.4		125.5		126.1		132.1
(2)-OCH_3	4.08 s	56.2	4.05 s	55.9	4.00 s	56.1	3.95 s	56.2
(4)-OCH_3	3.84 s	62.1	3.95 s	59.5	3.89 s	60.2	3.76 s	61.7
(6)-OCH_3								
(7)-OCH_3			3.95 s	55.1	3.97 s	55.9		
(8)-OCH_3					3.94 s	61.4		
(3)-OH	5.95 s		5.97 s				5.52 s	
(5)-OH	10.27 s						8.55 s	

Table S2: NMR data of compounds **5**, **6** and **7** in $CDCl_3$ (600 MHz, δ ppm)

	**Compound 5**		**Compound 6**		**Compound 7**	
	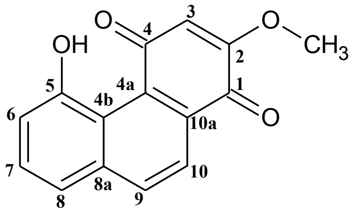		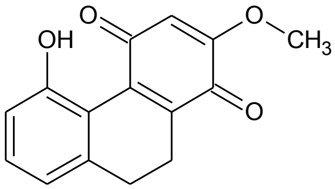		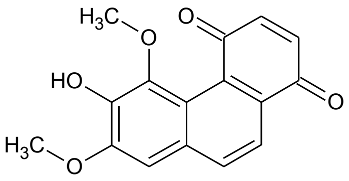	
	**^{1}H (*J* in Hz)**	**^{13}C**	**^{1}H (*J* in Hz)**	**^{13}C**	**^{1}H (*J* in Hz)**	**^{13}C**
1	-	180.0	-	180.3	-	185.0
2	-	158.9	-	158.1	6.84 d (10.2)	135.2
3	6.29 s	111.4	6.04 s	107.9	7.07 d (10.2)	140.3
4	-	191.6	-	191.1	-	186.1
4a	-	129.9	-	138.5	-	131.7
4b	-	121.4	-	116.9	-	119.6
5	-	155.7	-	154.8	-	142.0
6	7.25 dd (7.8/1.4)	117.4	6.91 dd (7.6/1.2)	118.7	-	140.4
7	7.59 t (7.8)	130.9	7.22 t (7.6)	131.8	-	150.5
8	7.43 dd (7.8/1.4)	120.8	6.78 dd (7.6/1.2)	119.7	6.99 s	102.3
8a	-	138.9	-	140.0	-	131.8
9	8.16 d (8.7)	137.2	2.70 (2H) m	27.8	7.89 d (8.4)	132.2
10	8.19 d (8.7)	121.8	2.65 (2H) m	20.7	7.95 d (8.4)	120.2
10a	-	132.4	-	142.6	-	131.3
(2)-OCH_3	3.98 s	56.4	3.90 s	55.9	-	-
(5)-OH	12.22 s	-	9.81 s	-	-	-
(6)-OH		-	-	-	6.21 brs	-
(5)-OCH_3		-	-	-	3.93 s	60.2
(7)-OCH_3		-	-	-	4.07 s	56.1

Table S3: NMR data of compounds **8**, **9**, and **10** in $CDCl_3$ (600 MHz, δ ppm).

	**Compound 8**		**Compound 9**		**Compound 10**	
	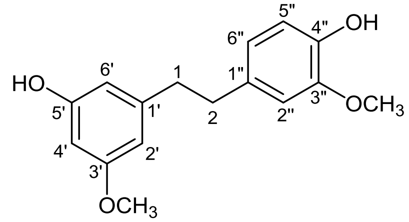		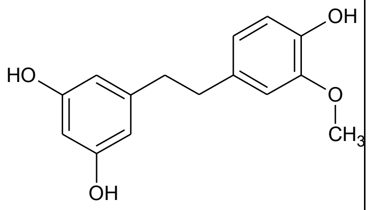		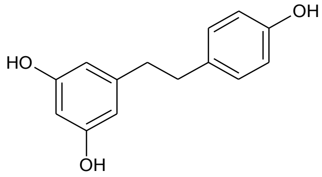	
	**^{1}H (*J* in Hz)**	**^{13}C**	**^{1}H (*J* in Hz)**	**^{13}C**	**^{1}H (*J* in Hz)**	**^{13}C**
1	2.81 m	37.4	2.79 m	37.0	2.77 m	36.4
2	2.83 m	38.4	2.82 m	37.6	2.82 m	37.5
1′	-	144.7	-	144.9	-	144.8
2′	6.25 brds	108.2	6.22	108.1	6.23 d (2.2)	107.8
3′	-	161.0	-	156.5	-	155.2
4′	6.25 brds	99.2	6.19 t (2.2)	100.3	6.18 t (2.2)	100.2
5′	-	156.8	-	156.5	-	155.2
6′	6.32 t (1.7)	107.0	6.22 m	108.1	6.23 d (2.2)	107.8
1′′	-	133.8	-	133.4	-	133.6
2′′	6.62 d (2.0)	111.4	6.61 d (2.0)	111.2	7.03 d (8.5)	129.1
3′′	-	146.5	-	146.4	6.74 d (8.5)	114.9
4′′	-	143.9	-	143.8	-	154.1
5′′	6.83 d (8.3)	114.4	6.82 d (7.9)	114.3	6.74 d (8.5)	114.9
6′′	6.68 dd (8.3/2.0)	121.2	6.67 dd (7.9/2.0)	121.2	7.03 d (8.5)	129.1
(3′)-OCH_3	3.75 s	55.4	-	-		
(3′′)-OCH_3	3.84 s	56.0	3.84 s	55.6		
(3′)-OH	-		4.75 brds			
(5′)-OH	4.63 s		4.75 brds			
(4′′)-OH	5.46 s		5.45 s			

Table S4: NMR data of compounds **11** and **12** in $CDCl_3$ (600 MHz, δ ppm).

	**Compound 11**		**Compound 12**	
	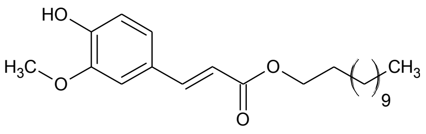		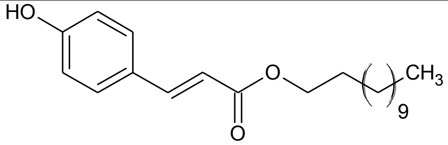	
	**^{1}H (*J* in Hz)**	**^{13}C**	**^{1}H (*J* in Hz)**	**^{13}C**
1	-	127.2	-	127.7
2	7.04 d (1.9)	108.9	7.43 d (8.7)	129.9
3	-	146.7	6.83 d (8.7)	115.9
4	-	147.9	-	157.6
5	6.92 d (8.1)	114.2	6.83 d (8.7)	115.9
6	7.07 dd (8.1/1.9)	122.6	7.43 d (8.7)	129.9
7	7.61 d (15.8)	144.1	7.62 d (15.8)	144.3
8	6.29 d (15.8)	115.4	6.30 d (15.8)	116.1
9	-	167.6	-	167.5
1′	4.19 t (6.8) 2H	64.1	4.18 t (6.8) 2H	64.6
2′	1.69 m 2H	28.0	1.69 m 2H	28.3
3′	1.39 m 2H	25.6	1.39 m 2H	25.9
4′-11′	1.25-1.27 m	22.1-31.2	1.25-1.27 m	22.1-31.2
12′	0.88 t (6.9)	13.4	0.88 t (6.9)	14.1
$-OCH_3$	3.93 s	55.3	-	

Table S5: NMR data of compounds **13** in $CDCl_3$ (600 MHz, δ ppm).

	**Compound 13**	
	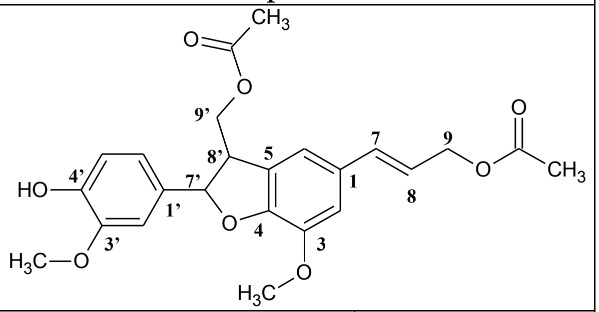	
	**^{1}H (*J* in Hz)**	**^{13}C**
1	-	130.2
2	6.88 m	110.2
3	-	144.0
4	-	147.8
5	-	127.2
6	6.88 m	114.8
7	6.60 d (15.9)	133.9
8	6.15 dt (15.9/6.5)	120.7
9	4.71 d (6.5)	64.6
1′	-	131.8
2′	6.88 m	108.2
3′	-	146.2
4′	-	145.7
5′	6.88 m	113.8
6′	6.88 m	119.0
7′	5.47 d (7.3)	88.2
8′	3.77 m	49.8
9′	4.43 dd (11.0/5.6)<br>4.31 dd (11.0/7.3)	64.7
(3)-OCH_3	3.90 s	55.4
(3′)-OCH_3	3.86 s	55.4
(9)-CO$\underline{CH_3}$	2.02 s	20.1
(9′)-CO$\underline{CH_3}$	2.10 s	20.4
(9)-$\underline{C}OCH_3$	-	170.2
(9′)-$\underline{C}OCH_3$	-	170.4
(4′)-OH	5.61 s	-

Table S6: NMR data of compounds **14** in $CDCl_3$ (600 MHz, δ ppm).

	**Compound 14**	
	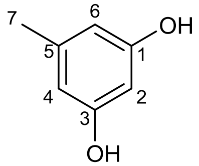	
	**^{1}H (*J* in Hz)**	**^{13}C**
1	-	156.9
2	6.17 t (*J* = 2.3 Hz)	100.1
3	-	156.9
4	6.24 d (*J* = 2.2 Hz)	108.9
5	-	141.1
6	6.24 d (*J* = 2.2 Hz)	108.9
7	2.24 s (3H)	21.5

Table S7: NMR data of compounds **15** and **16** in $CDCl_3$ (600 MHz, δ ppm).

	Compound 15		Compound 16	
	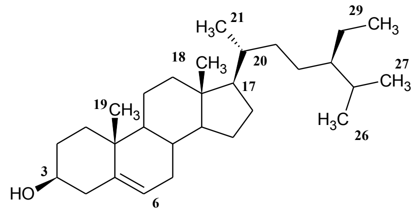		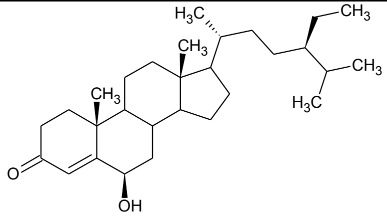	
	^{1}H (*J* in Hz)	^{13}C	^{1}H (*J* in Hz)	^{13}C
1	1.89 m<br>1.15 m	37.2	2.03 m<br>1.71 ddd (15.0/14.2/4.2)	37.25
2	1.85 m<br>1.56 m	31.6	2.52 ddd (17.1/15.0/5.0)<br>2.37 ddd (17.1/4.2/1.0)	34.2
3	3.53 m	71.8	-	200.3
4	2.28 m	42.3	5.82 s	126.6
5	-	140.7	-	168.4
6	5.35 m	121.7	4.34 brds	73.4
7	1.53 m	31.9	2.00 m / 1.24 m	38.6
8	1.93 m	31.9	1.95 m	30.1
9	0.98 m	50.1	0.90 m	53.6
10	-	36.5	-	37.9
11	1.51 m	21.1	1.49 m (2H)	21.0
12	2.03 m / 1.19 m	39.7	2.03 m / 1.15 m	39.7
13	-	42.3	-	42.4
14	1.00 m	56.7	1.01 m	55.9
15	1.61 m / 1.13 m	24.3	1.61 m / 1.13 m	24.5
16	1.86 m / 1.35 m	28.2	1.86 m / 1.30 m	28.3
17	1.10 m	56.0	1.12 m	56.2
18	0.68 s	11.8	0.74 s	11.6
19	1.00 s	19.4	1.38 s	19.6
20	1.37 m	36.1	1.35 m	36.3
21	0.92 d (6.5)	18.8	0.92 d (6.6)	18.7
22	1.35 m / 1.04 m	33.9	1.31 m / 1.01 m	34.1
23	1.20 m	26.0	1.16 m	26.3
24	1.97 m	45.8	0.93 m	45.9
25	1.70 m	29.1	1.66 m	29.4
26	0.84 d (6.5)	19.8	0.81 d (6.8)	19.6
27	0.81 d (6.5)	19.0	0.84 d (6.9)	19.1
28	1.27 m / 1.21 m	23.0	1.27 m / 1.21 m	23.2
29	0.85 t (7.5)	12.0	0.85 t (7.5)	11.9

Table S8: P-values after one-way Anova by Dunnett's multiple comparisons test against the positive control on collagenase assay.

**Dunnett's multiple comparisons test**	**Significant**	**Summary**	**P Value**
Phosphoramidon vs. Flowers DCM	Yes	****	<0.0001
Phosphoramidon vs. Flowers EtoAc	Yes	****	<0.0001
Phosphoramidon vs. Flowers EtOH: H2O	No	ns	0.0848
Phosphoramidon vs. Leaves DCM	No	ns	0.1652
Phosphoramidon vs. Leaves EtoAc	Yes	****	<0.0001
Phosphoramidon vs. Leaves EtOH: H2O	Yes	****	<0.0001
Phosphoramidon vs. Pseudobulbs DCM	Yes	****	<0.0001
Phosphoramidon vs. Pseudobulbs EtoAc	Yes	****	<0.0001
Phosphoramidon vs. Pseudobulbs EtOH: H2O	No	ns	0.9959
Phosphoramidon vs. Roots DCM	No	ns	0.9994
Phosphoramidon vs. Roots EtoAc	Yes	****	<0.0001
Phosphoramidon vs. Roots EtOH: H2O	Yes	****	<0.0001

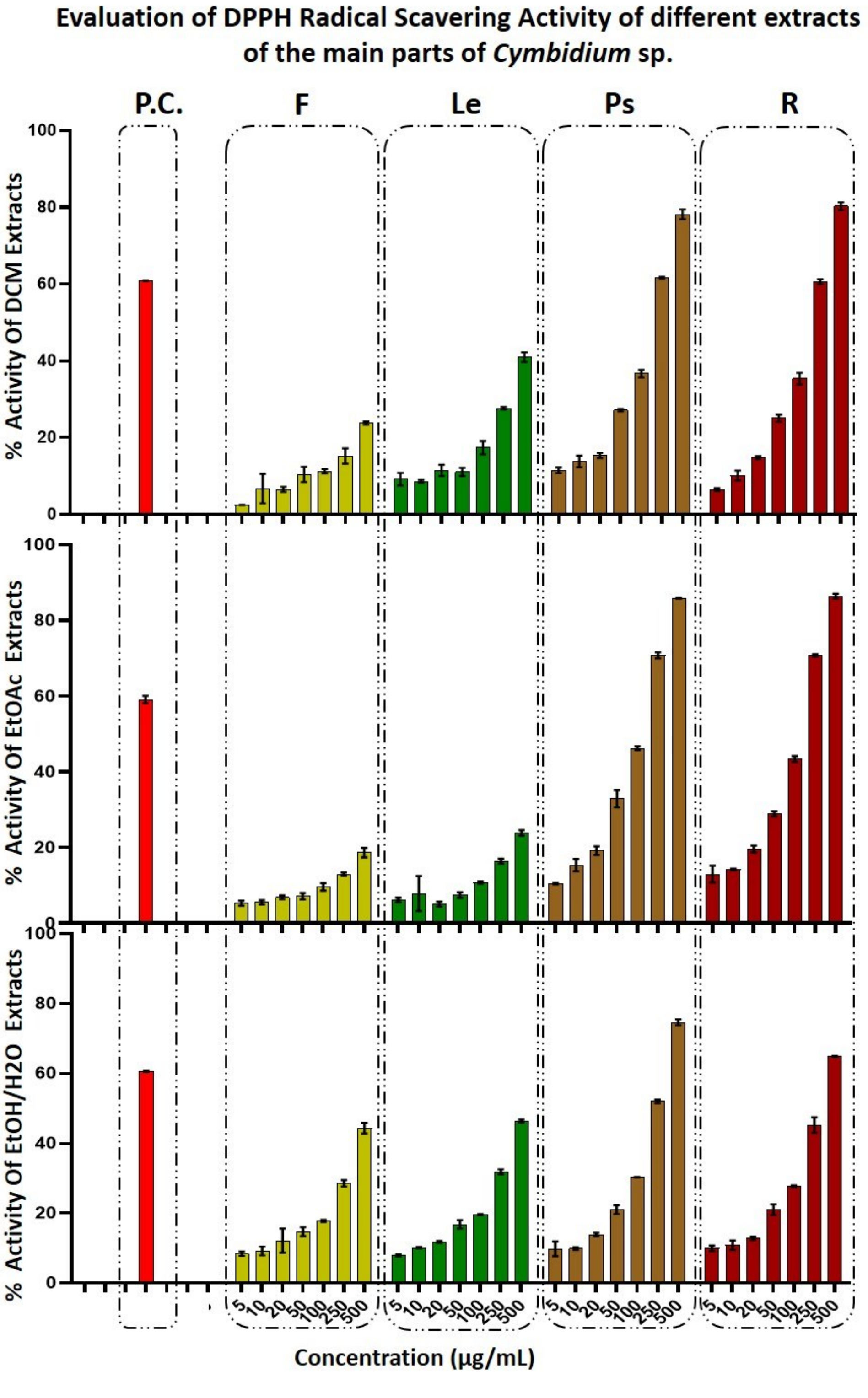

Figure S1: Determination of DPPH radical scavenging activity of DCM, EtOAc, and EtOH/H_2O extracts of Flowers, Leaves, Pseudobulbs, and Roots of *Cymbidium* sp. All experiments were performed in triplicate. Data are expressed as mean ±SD (n=3, $p<0.05$) for all tested dosages.

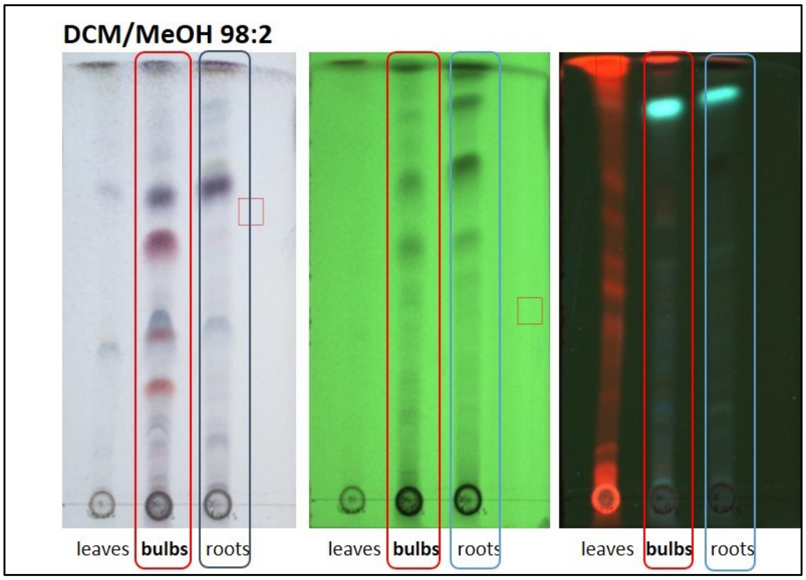

Figure S2: TLC chromatograms of EtOAc total extracts from *Cymbidium* sp. pseudobulbs (red) and roots (blue) in DCM/MeOH 98:2 (Left: vis, center: UV-254nm, right: UV-366nm).

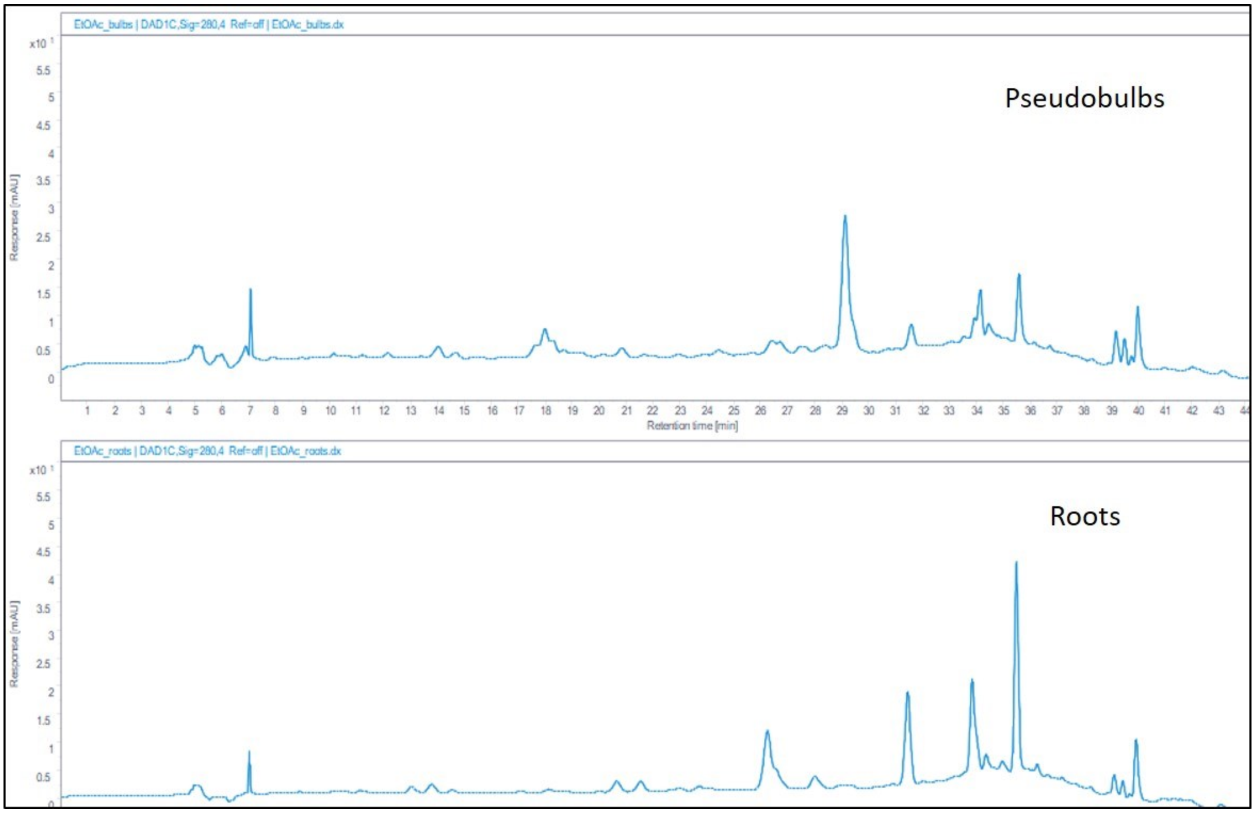

Figure S3. HPLC-DAD chromatogram of EtOAc total extracts from *Cymbidium* sp. bulbs and roots at 280 nm.

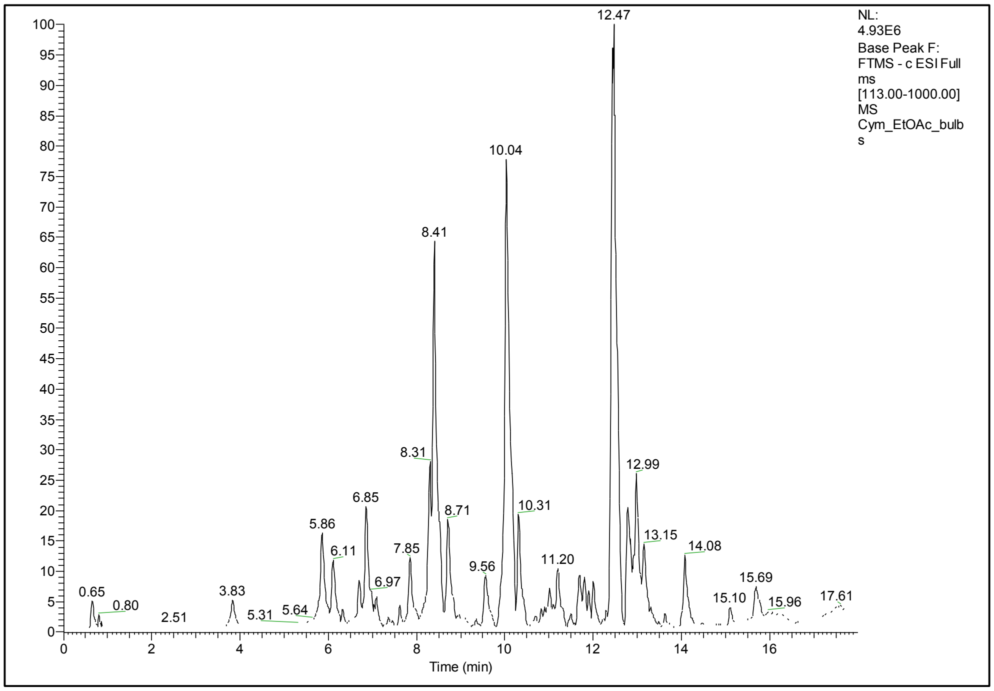

Figure S4: BP–UPLC-HRMS chromatogram of EtOAc total extract from *Cymbidium* sp. pseudobulbs.

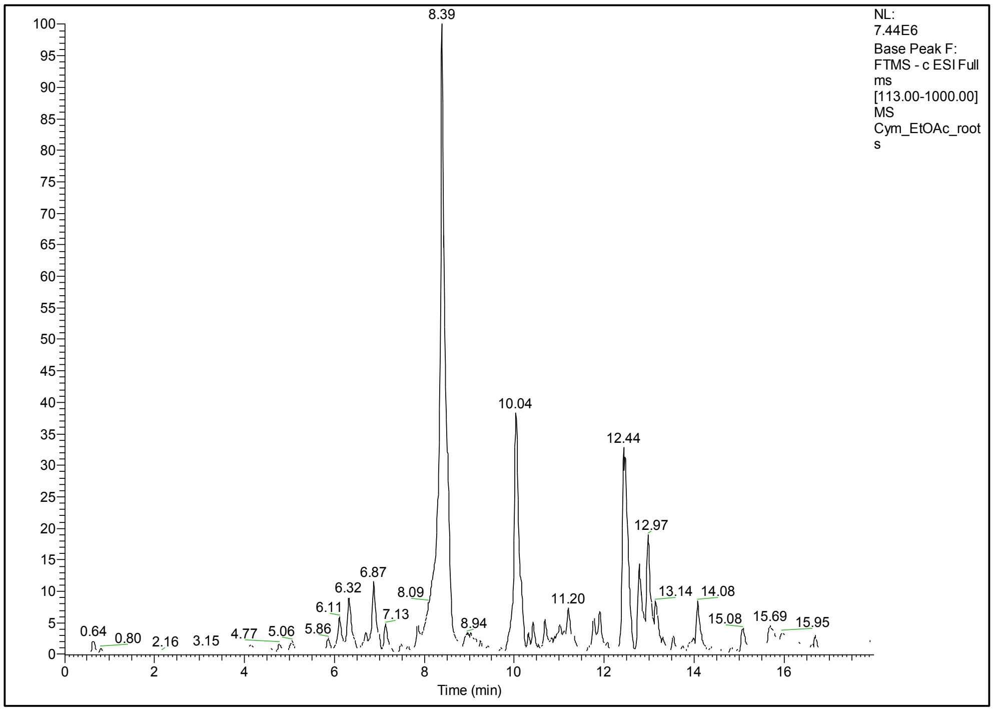

Figure S5: BP–UPLC-HRMS chromatogram of EtOAc total extract from *Cymbidium* sp. roots.

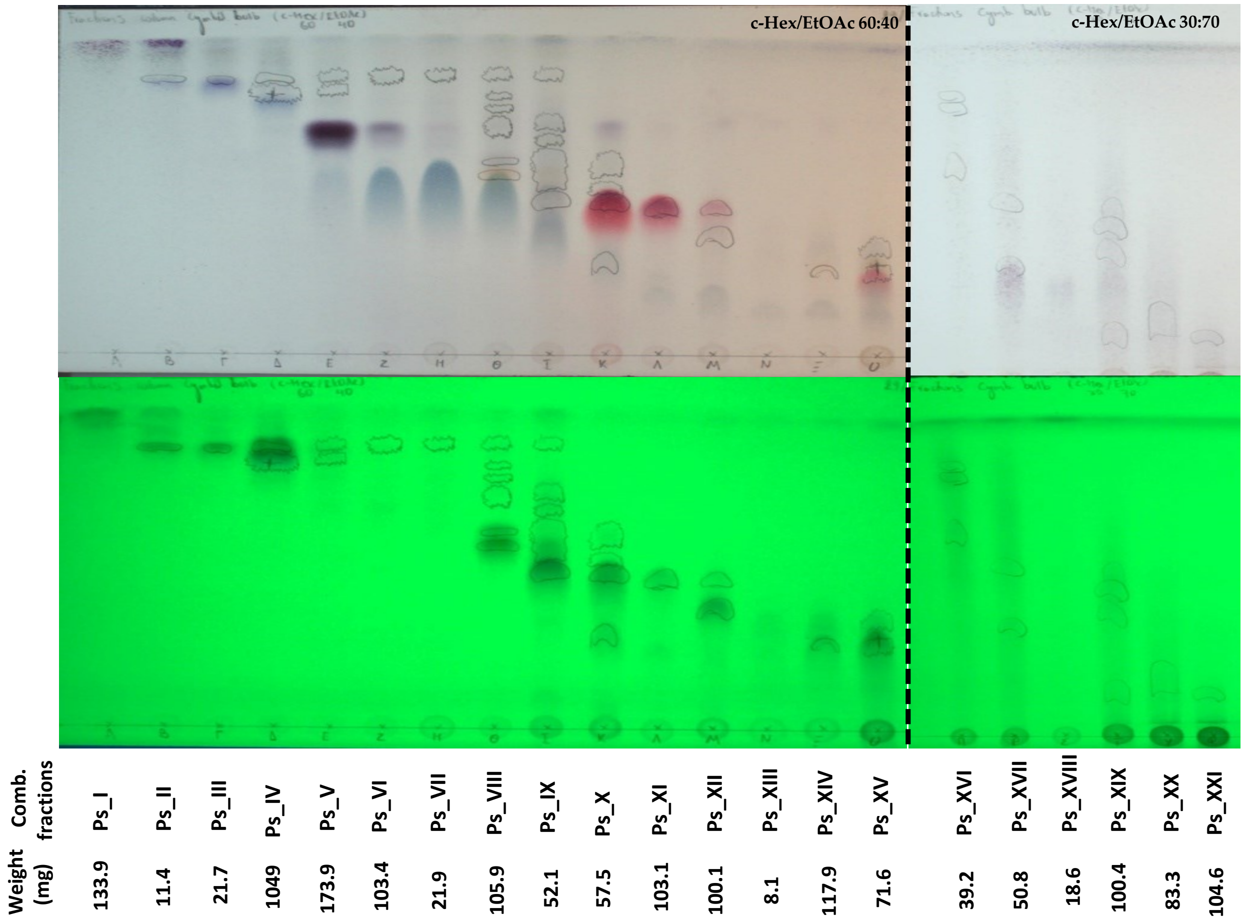

Figure S6: TLC chromatogram and weights of combined fractions obtained from the EtOAc pseudobulb extract through silica gel CC. Bottom: under UV lamp at 254 nm; Top: at Vis after spraying the plate with vanillin-sulfuric acid reagent and heating.

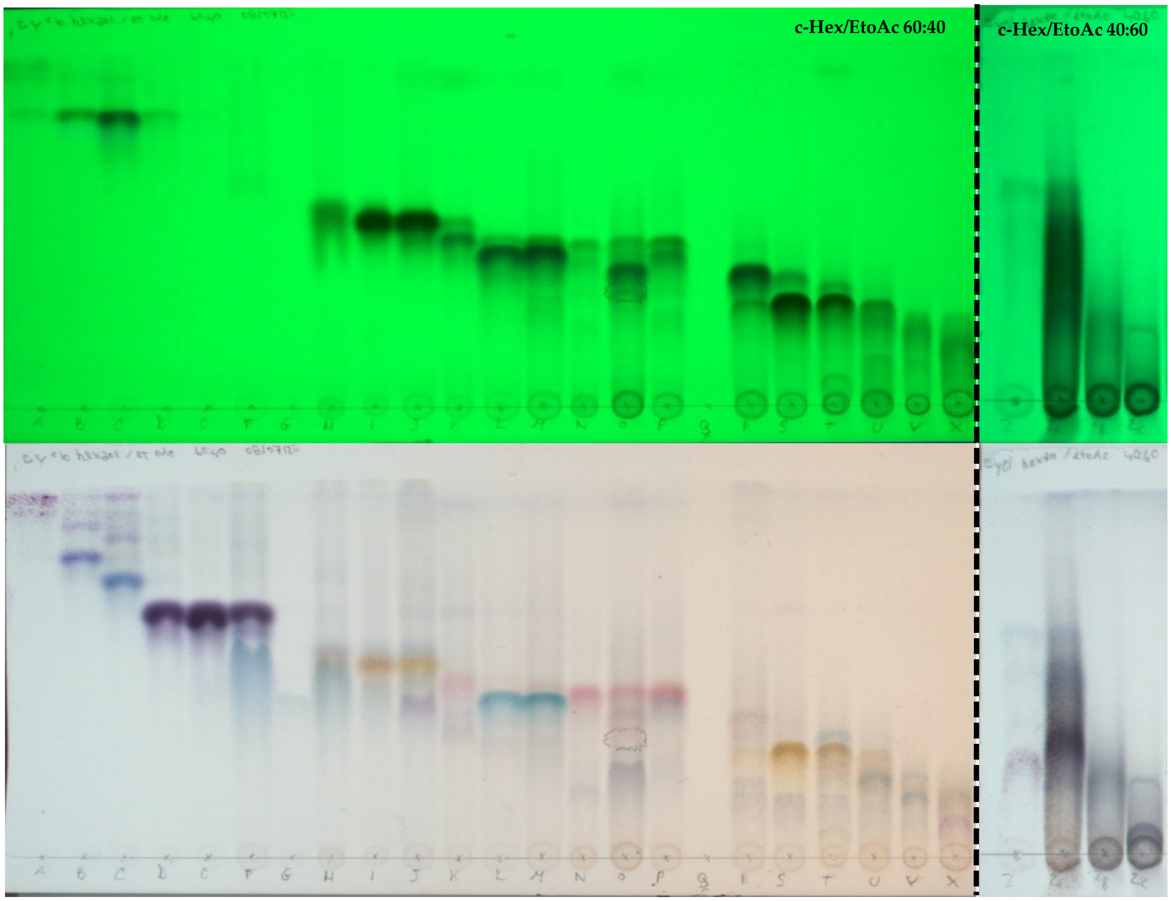

Comb. fractions	Weight (mg)
R_I	428.9
R_II	28.4
R_III	123.9
R_IV	114.0
R_V	259.8
R_VI	199.5
R_VII	40.5
R_VIII	60.4
R_IX	75.3
R_X	82.9
R_XI	66.7
R_XII	31.4
R_XIII	49.6
R_XIV	44.8
R_XV	19.9
R_XVI	67.5
R_XVII	53.0
R_XVIII	61.5
R_XIX	94.9
R_XX	52.2
R_XXI	138.5
R_XXII	34.6
R_XXIII	49.3
R_XXIV	229.4
R_XXV	69.6
R_XXVI	609.6
R_XXVII	28.1

Figure S7: TLC chromatogram and weights of combined fractions obtained from the EtOAc root extract through silica gel CC. Top: under UV lamp at 254 nm; Bottom: at Vis after spraying the plate with vanillin-sulfuric acid reagent and heating.

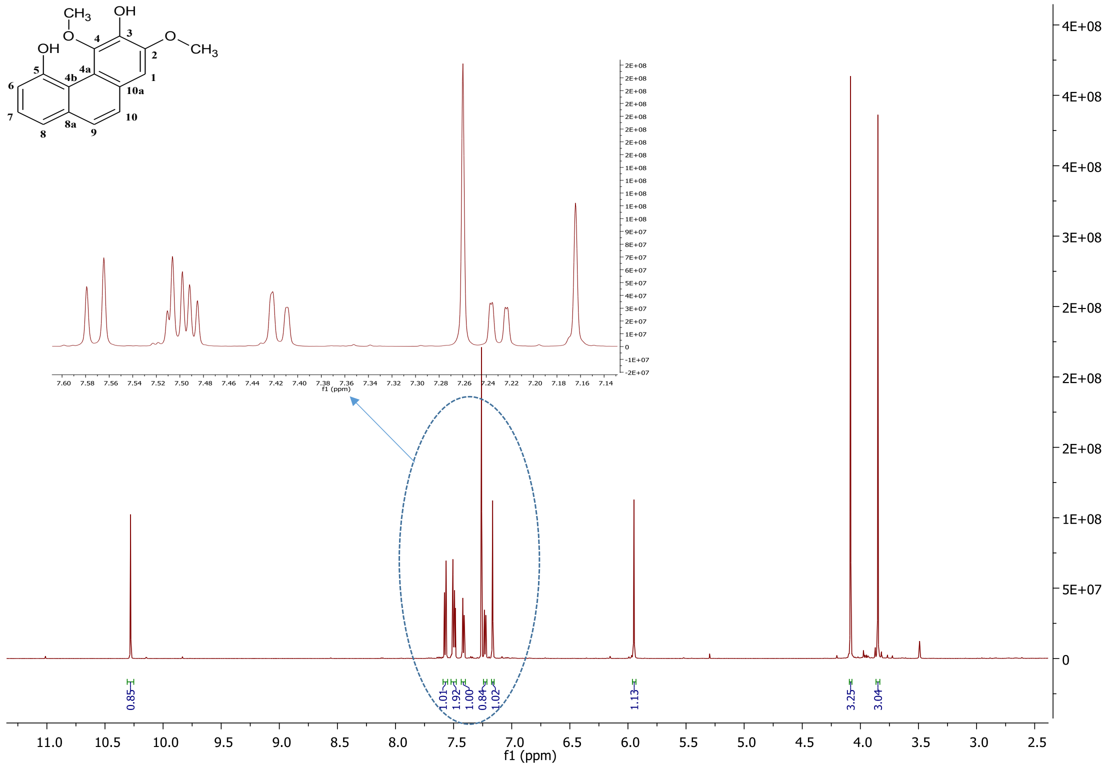

Figure S8a: ^{1}H-NMR spectrum of **compound 1** recorded in $CDCl_3$ at 600 MHz.

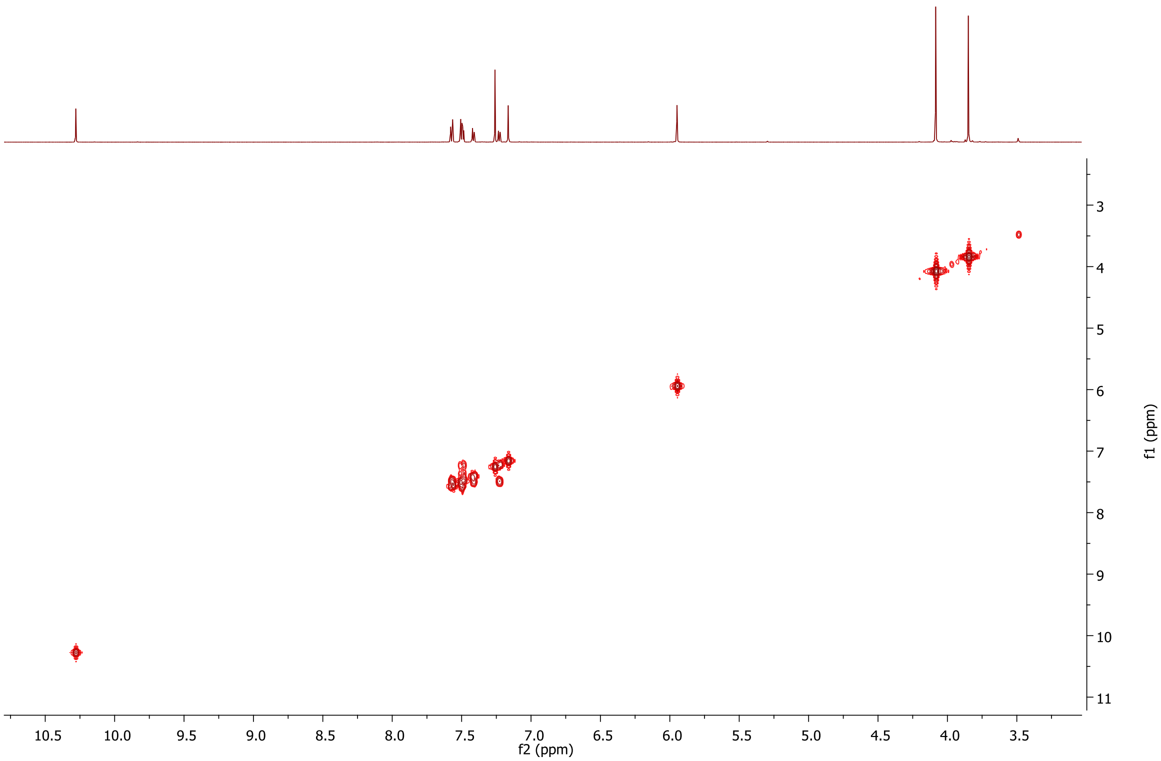

Figure S8b: COSY spectrum of **compound 1** recorded in $CDCl_3$ at 600 MHz.

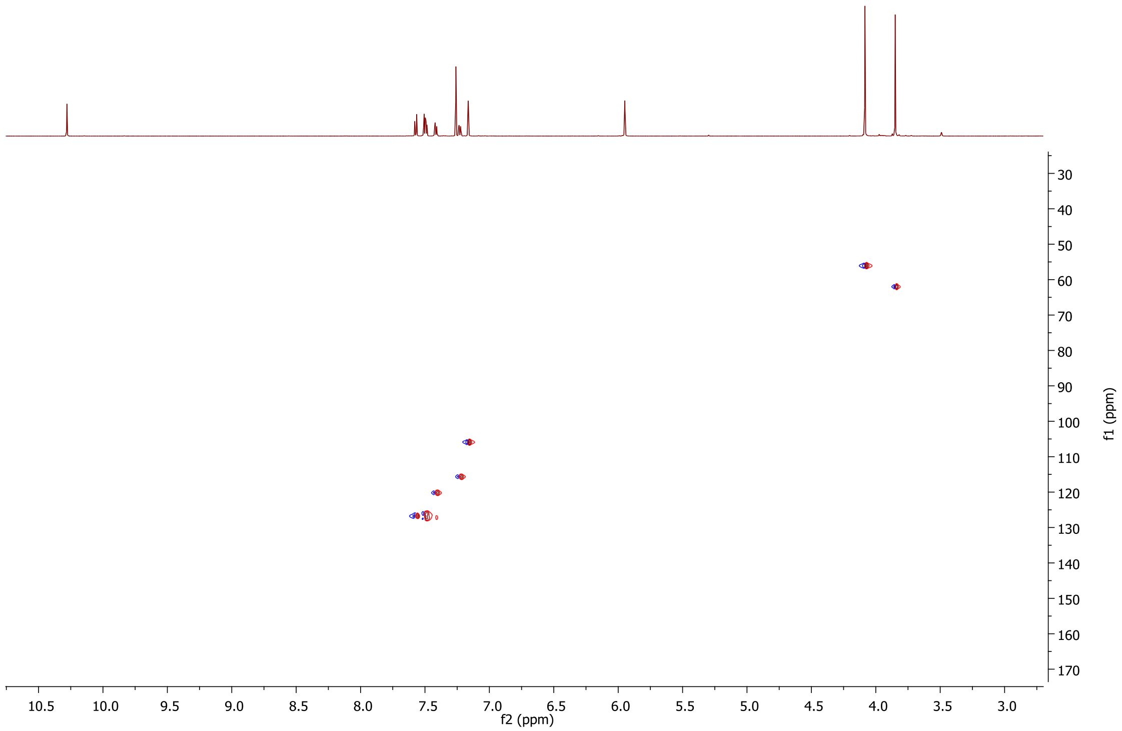

Figure S8c: HSQC-DEPT spectrum of **compound 1** recorded in $CDCl_3$ at 600 MHz.

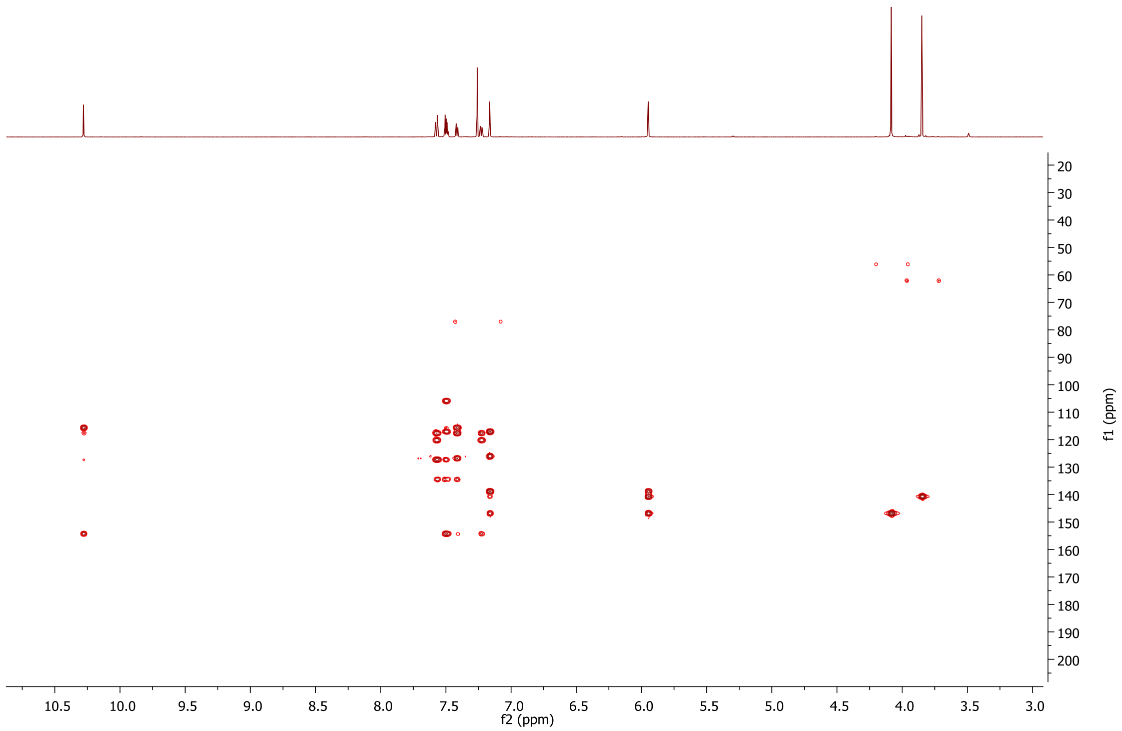

Figure S8d: HMBC spectrum of **compound 1** recorded in $CDCl_3$ at 600 MHz.

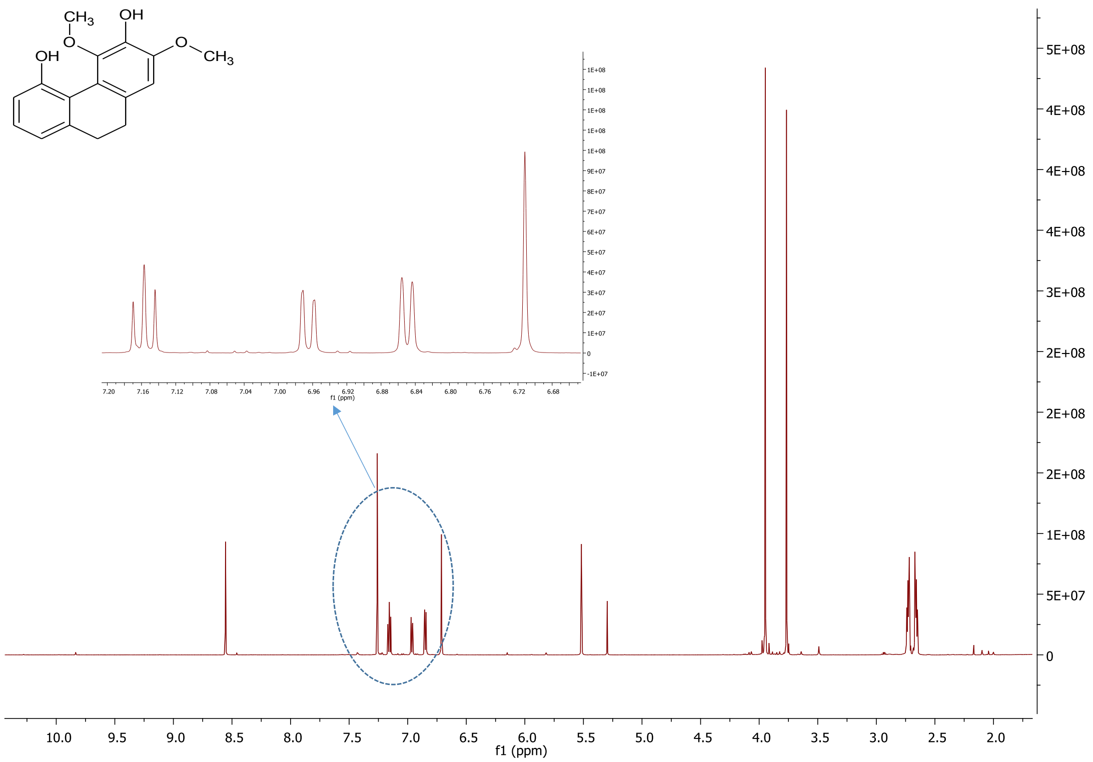

Figure S9a: ^{1}H-NMR spectrum of **compound 4** recorded in $CDCl_3$ at 600 MHz.

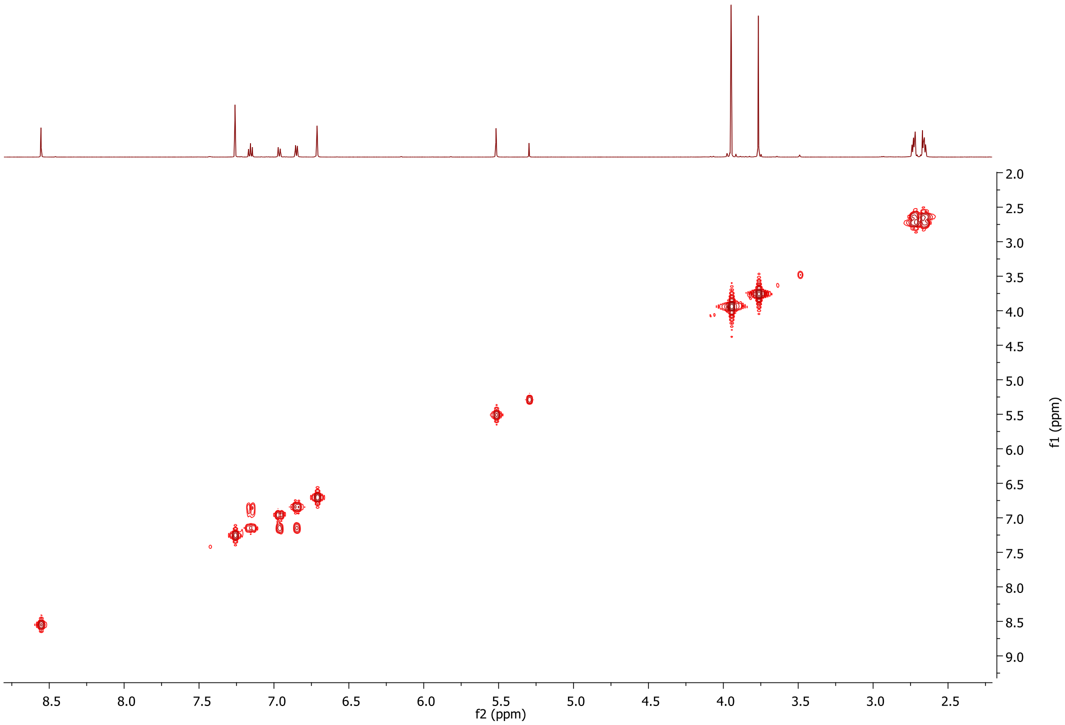

Figure S9b: COSY spectrum of **compound 4** recorded in $CDCl_3$ at 600 MHz.

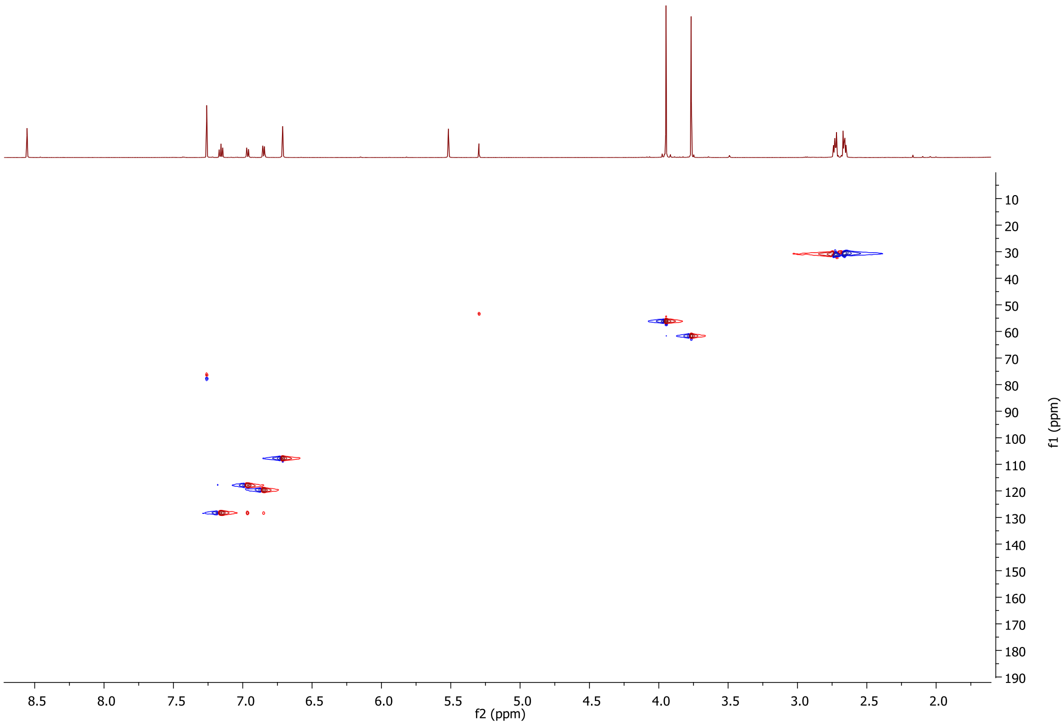

Figure S9c: HSQC-DEPT spectrum of **compound 4** recorded in $CDCl_3$ at 600 MHz.

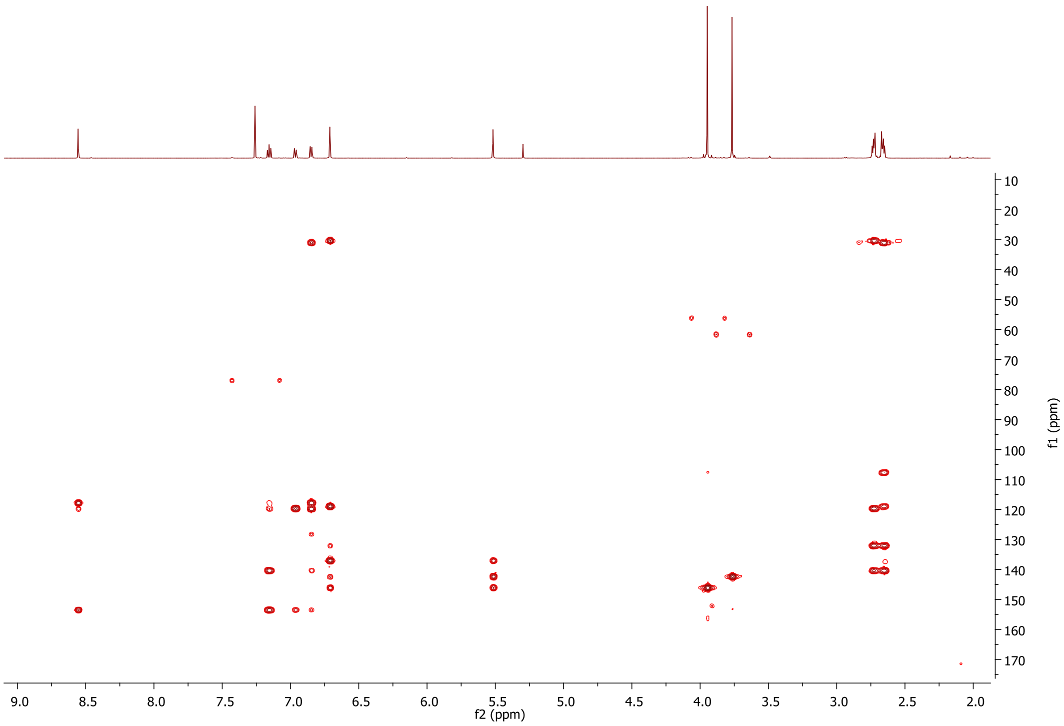

Figure S9d: HMBC spectrum of **compound 4** recorded in $CDCl_3$ at 600 MHz.

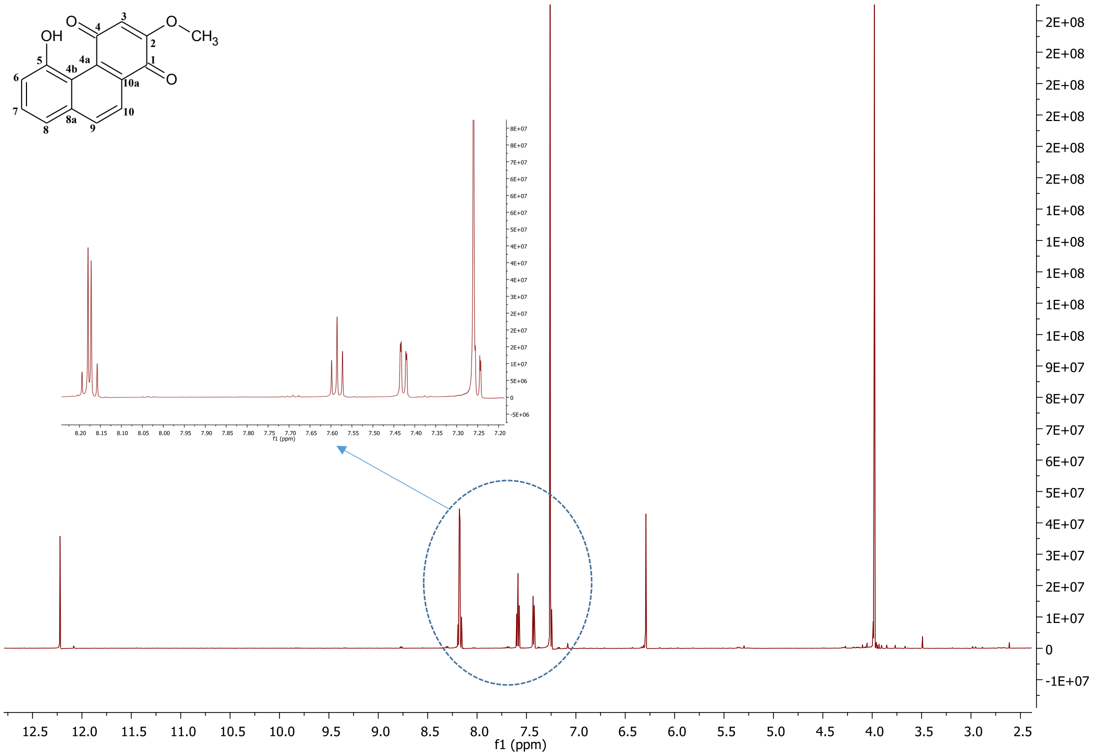

Figure S10a: ^{1}H-NMR spectrum of **compound 5** recorded in $CDCl_3$ at 600 MHz.

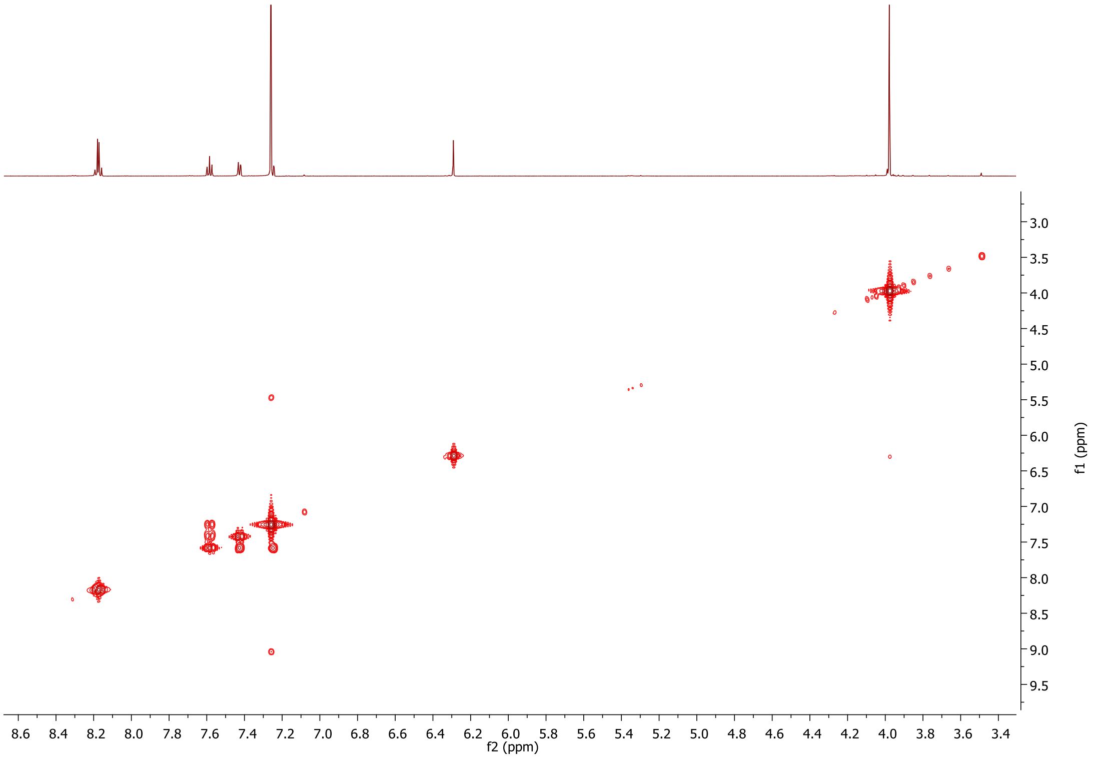

Figure S10b: COSY spectrum of **compound 5** recorded in $CDCl_3$ at 600 MHz.

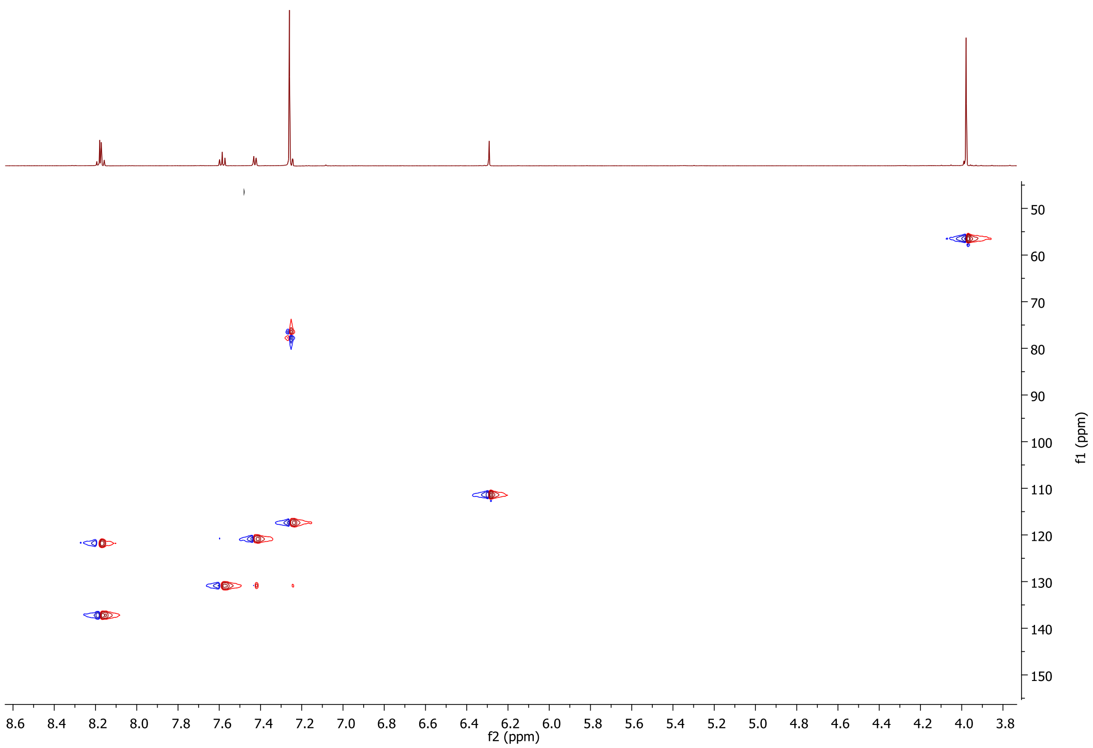

Figure S10c: HSQC-DEPT spectrum of **compound 5** recorded in $CDCl_3$ at 600 MHz.

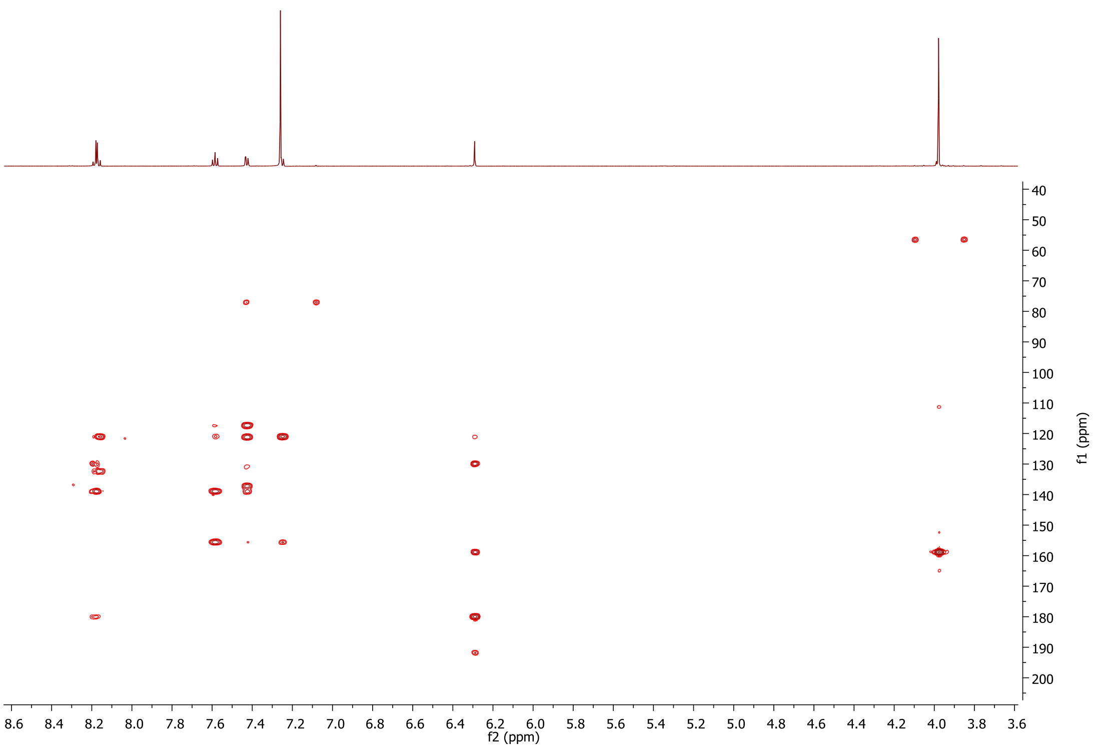

Figure S10d: HMBC spectrum of **compound 5** recorded in $CDCl_3$ at 600 MHz.

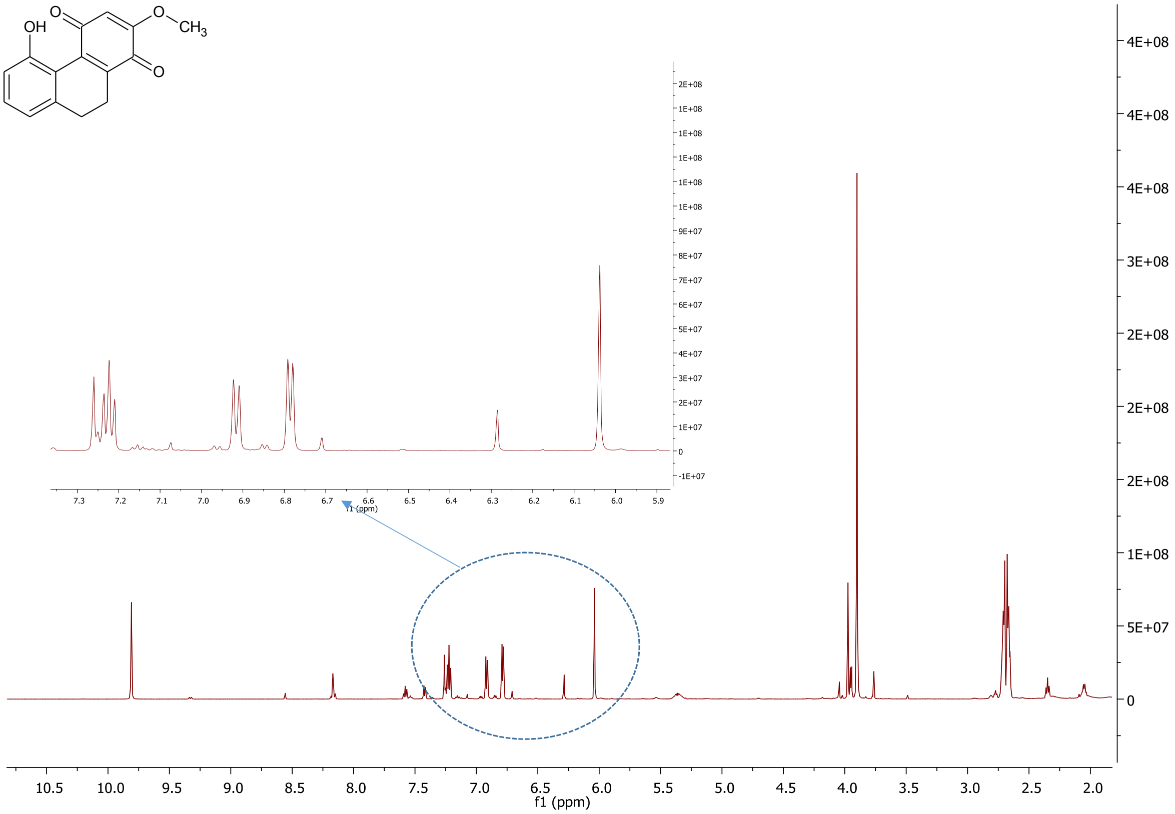

Figure S11: ^{1}H-NMR spectrum of **compound 6** recorded in $CDCl_3$ at 600 MHz.

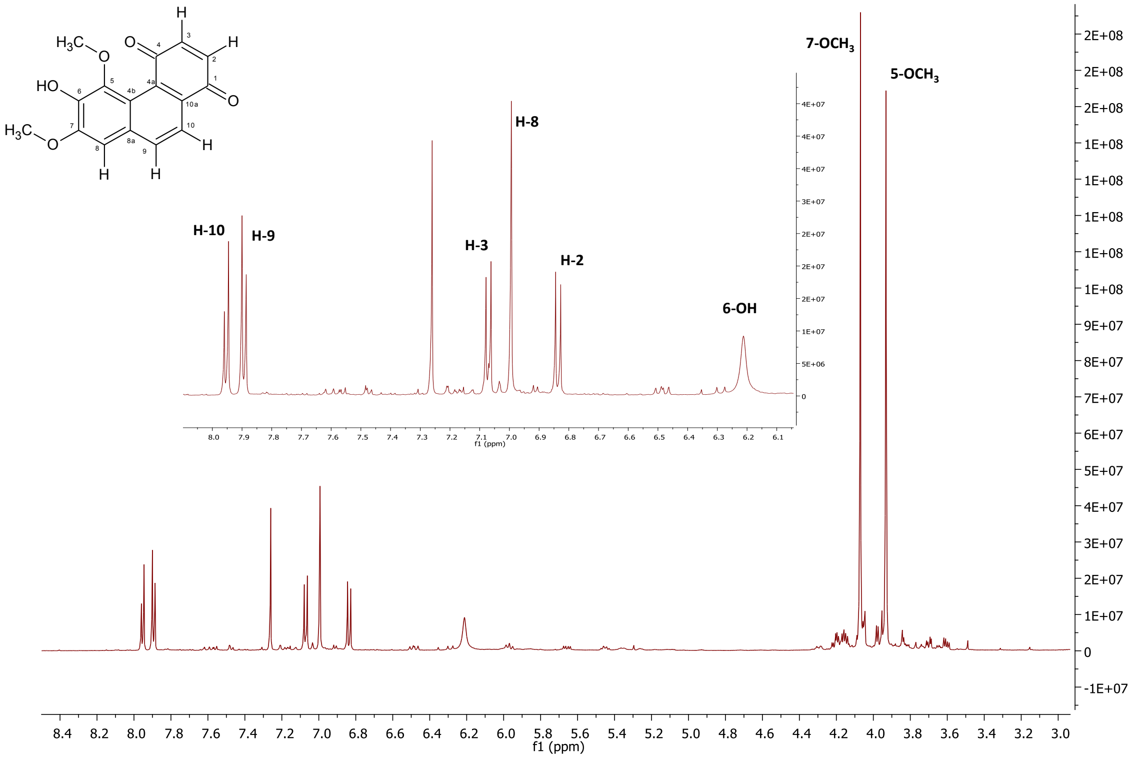

Figure S12a: ^{1}H-NMR spectrum of **compound 7** recorded in $CDCl_3$ at 600 MHz.

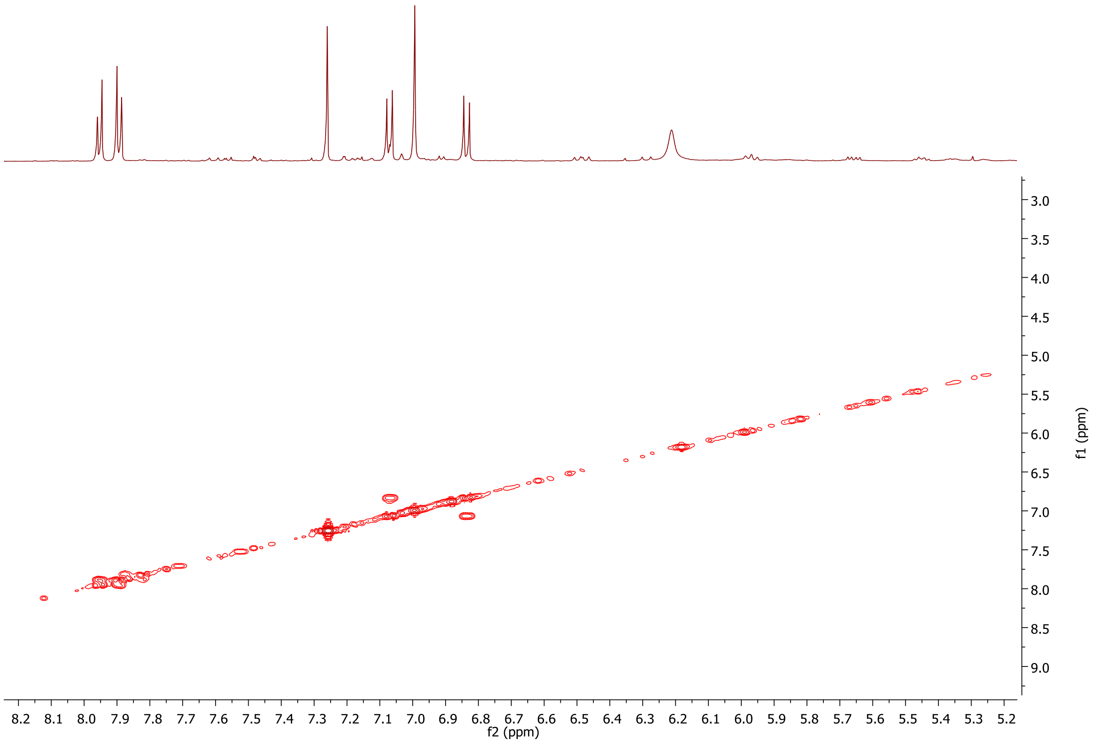

Figure S12b: COSY spectrum of **compound 7** recorded in $CDCl_3$ at 600 MHz.

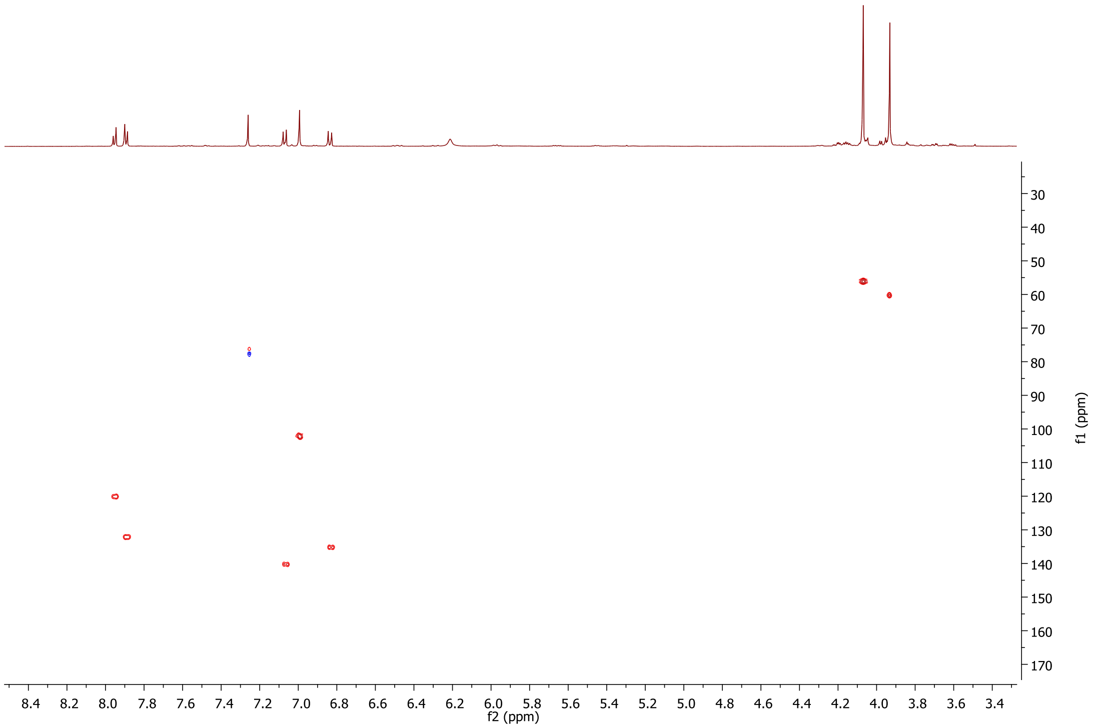

Figure S12c: HSQC-DEPT spectrum of **compound 7** recorded in $CDCl_3$ at 600 MHz.

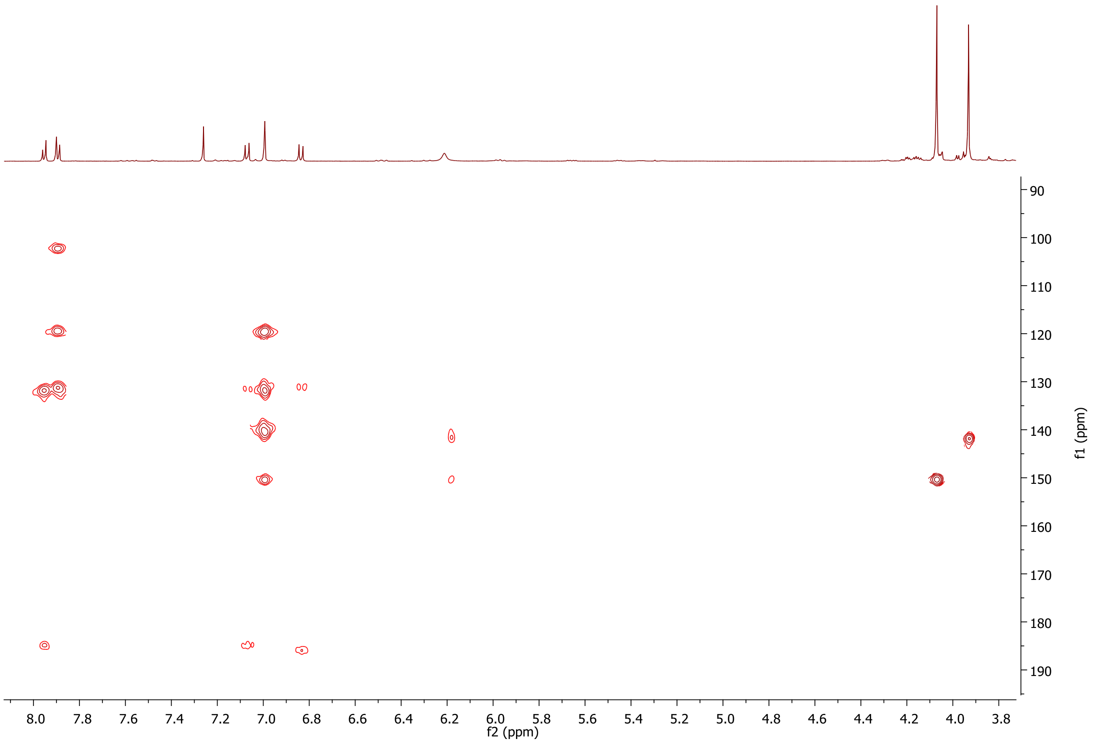

Figure S12d: HMBC spectrum of **compound 7** recorded in $CDCl_3$ at 600 MHz.

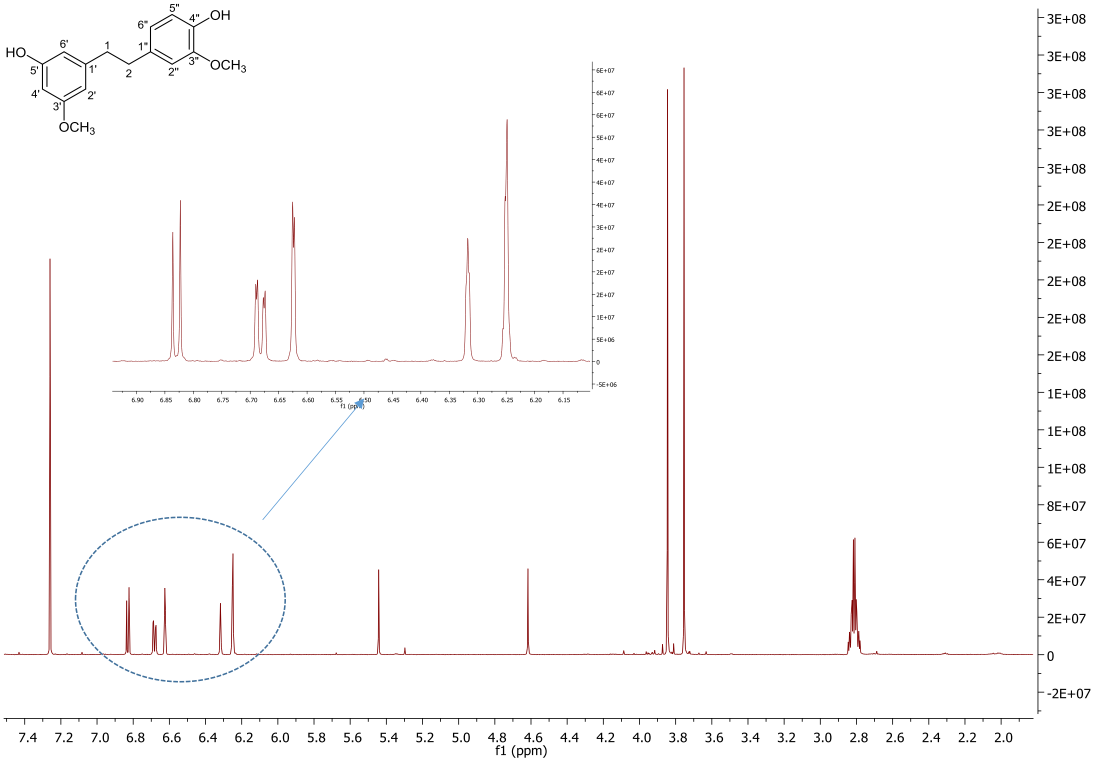

Figure S13a: ^{1}H-NMR spectrum of **compound 8** recorded in $CDCl_3$ at 600 MHz.

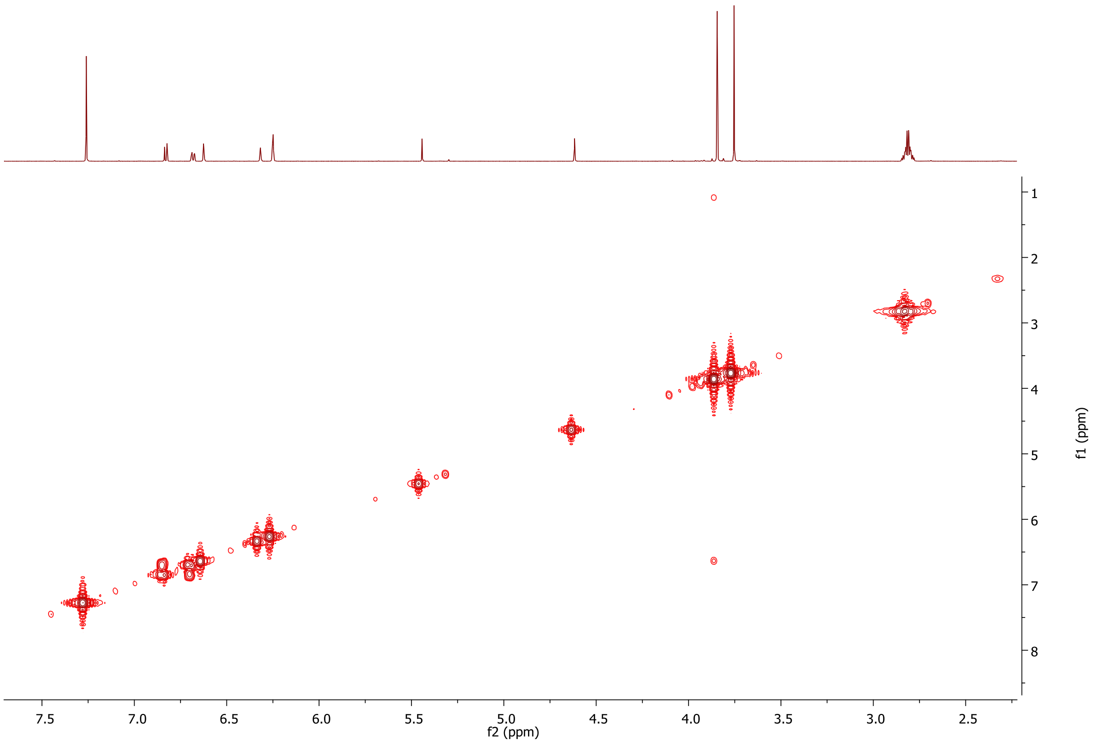

Figure S13b: COSY spectrum of **compound 8** recorded in $CDCl_3$ at 600 MHz.

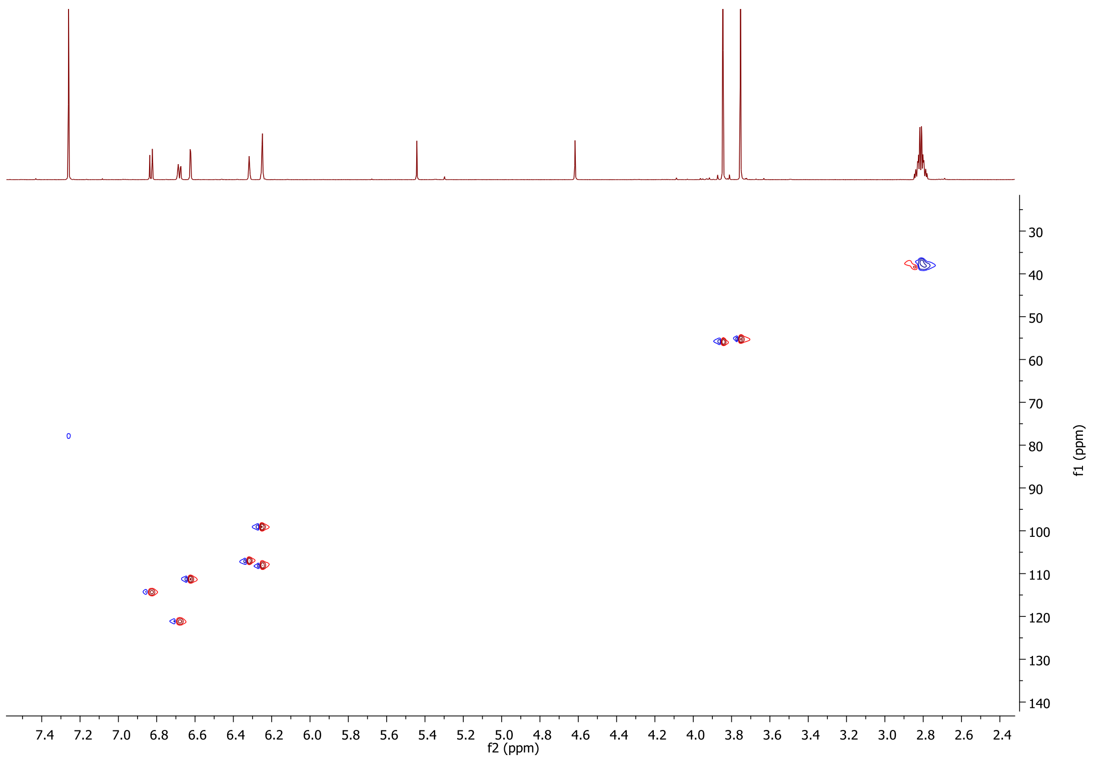

Figure S13c: HSQC-DEPT spectrum of **compound 8** recorded in $CDCl_3$ at 600 MHz.

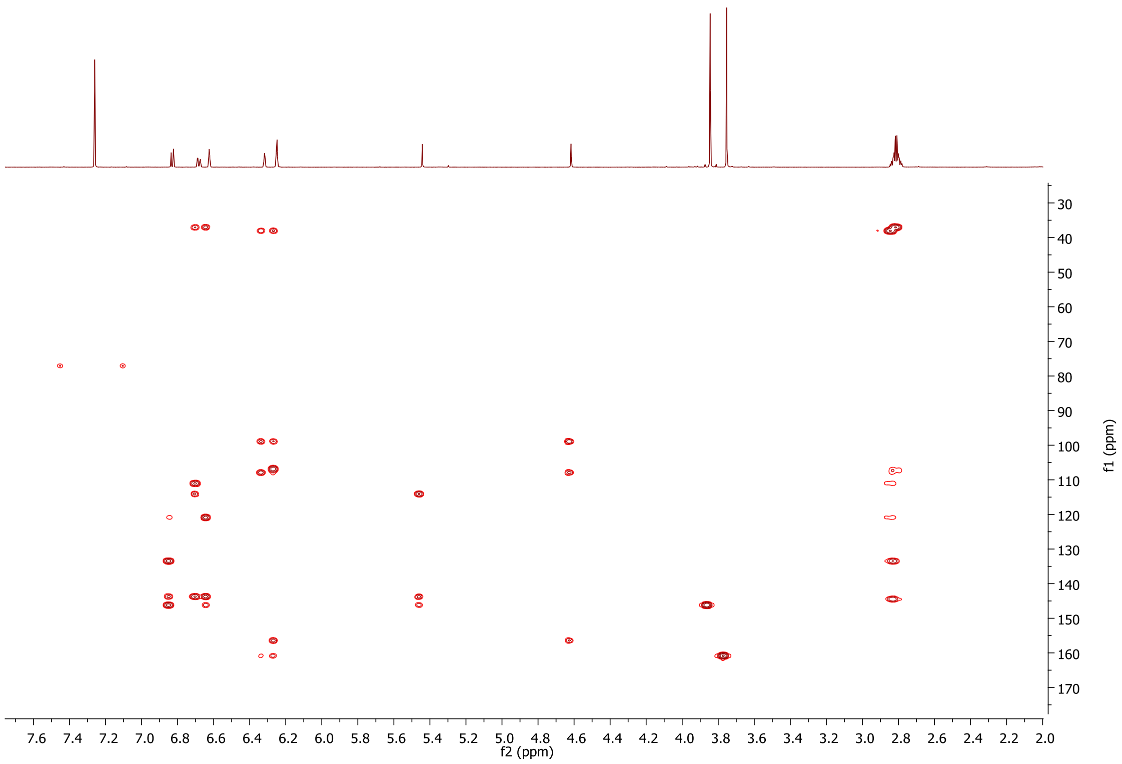

Figure S13d: HMBC spectrum of **compound 8** recorded in $CDCl_3$ at 600 MHz.

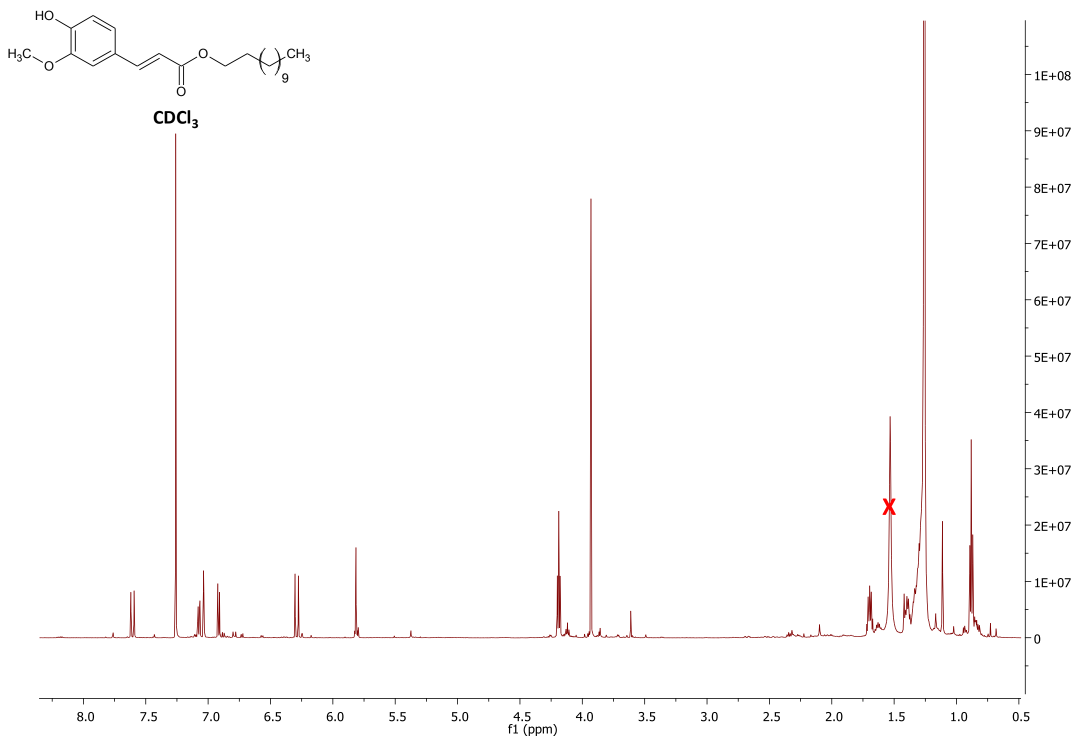

Figure S14a: ^{1}H-NMR spectrum of **compound 11** recorded in $CDCl_3$ at 600 MHz.

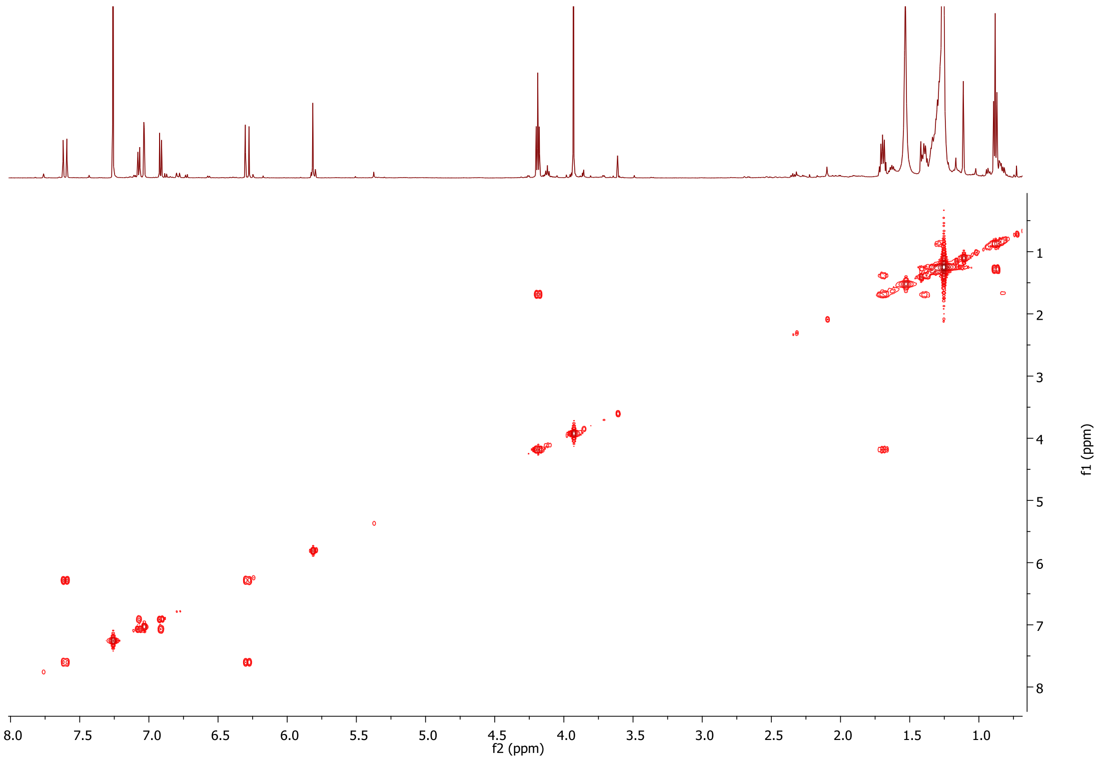

Figure S14b: COSY spectrum of **compound 11** recorded in $CDCl_3$ at 600 MHz.

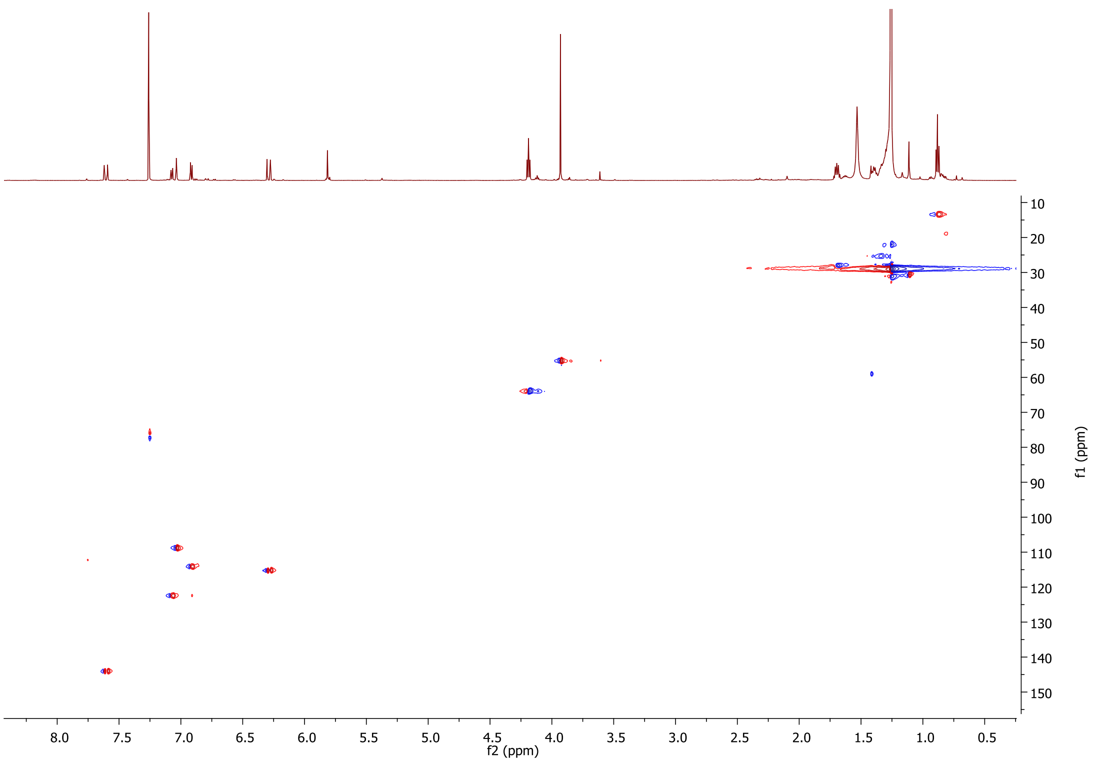

Figure S14c: HSQC-DEPT spectrum of **compound 11** recorded in $CDCl_3$ at 600 MHz.

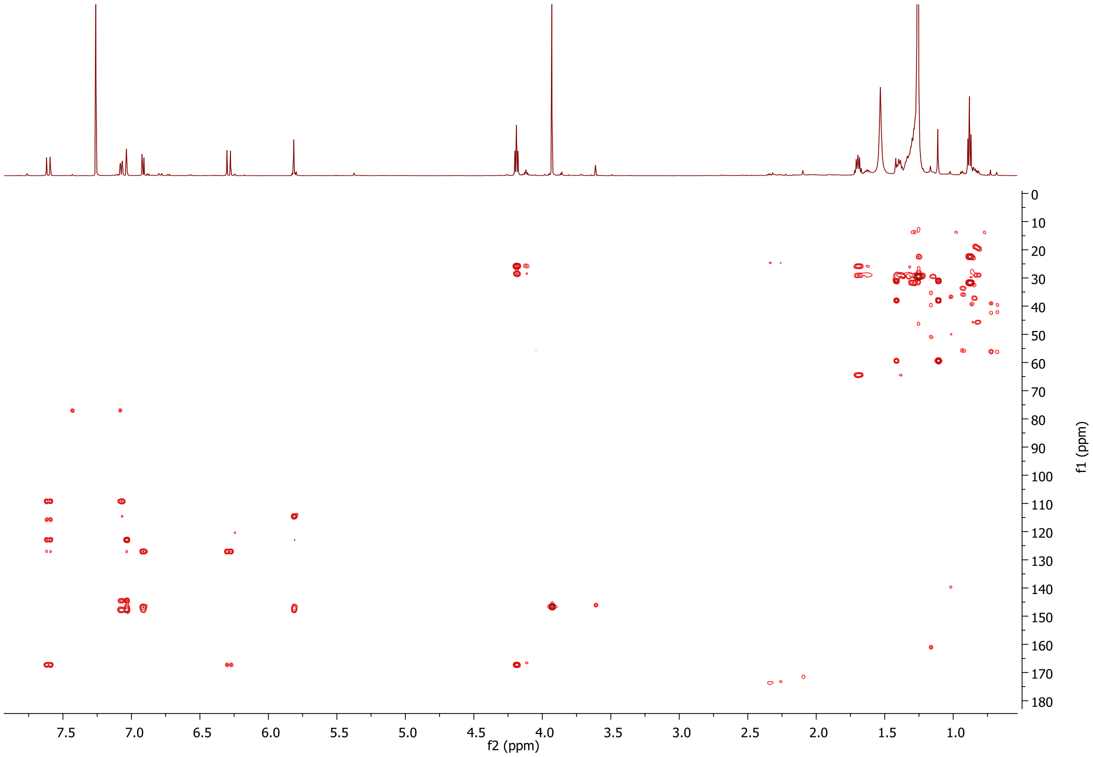

Figure S14d: HMBC spectrum of **compound 11** recorded in $CDCl_3$ at 600 MHz.

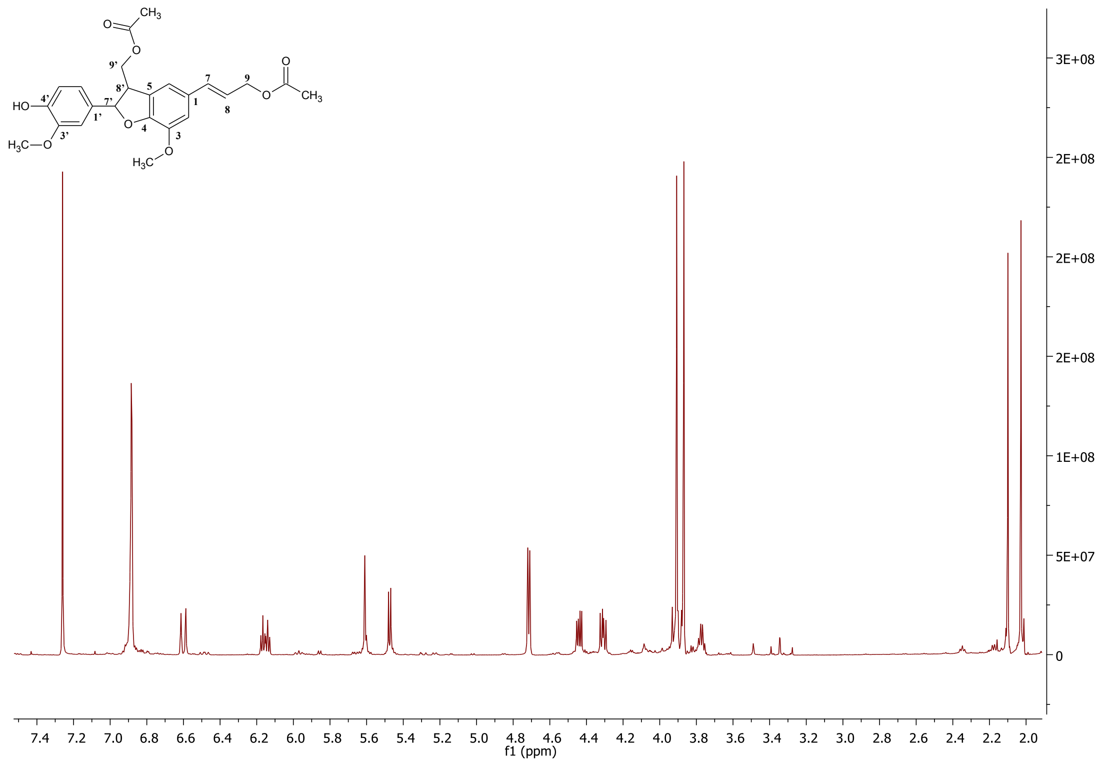

Figure S15a: ¹H-NMR spectrum of **compound 13** recorded in $CDCl_3$ at 600 MHz.

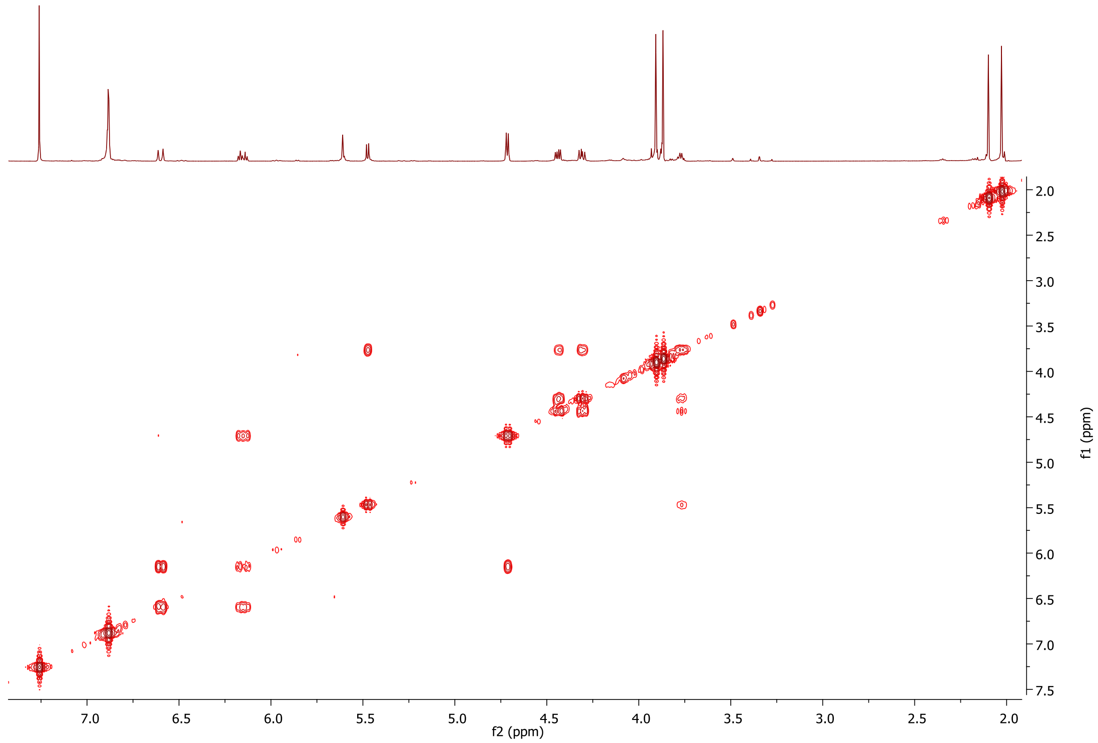

Figure S15b: COSY spectrum of **compound 13** recorded in $CDCl_3$ at 600 MHz.

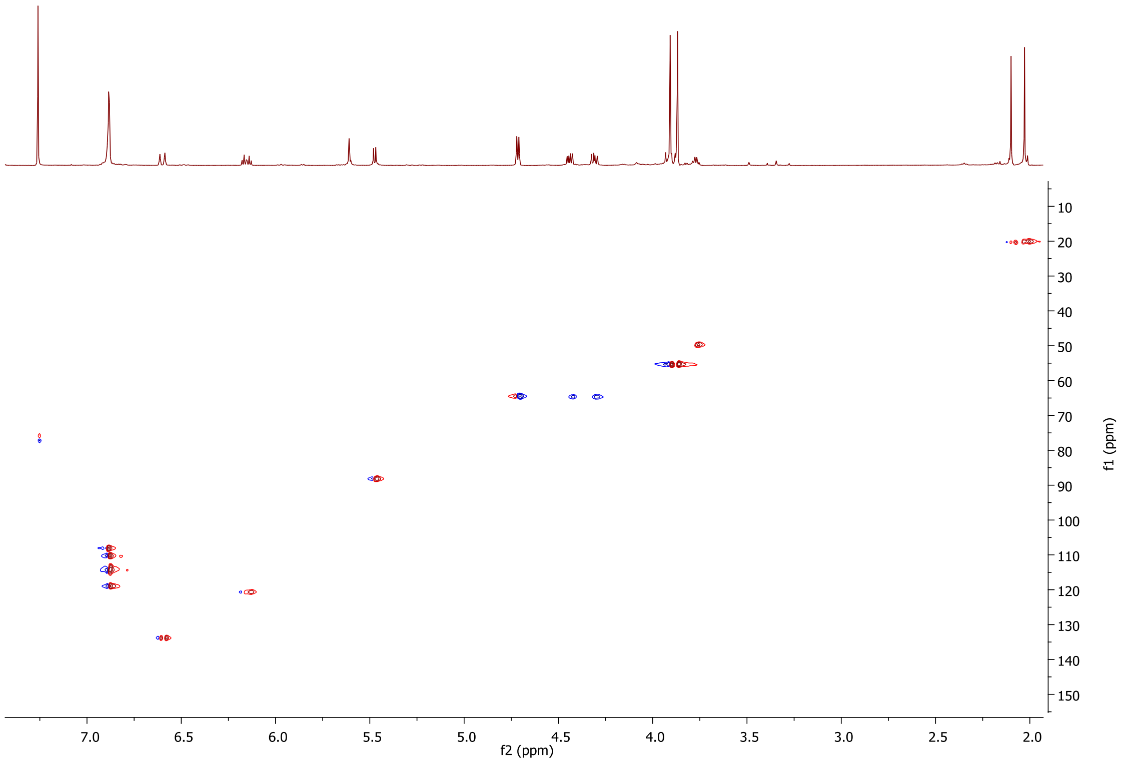

Figure S15c: HSQC-DEPT spectrum of **compound 13** recorded in $CDCl_3$ at 600 MHz.

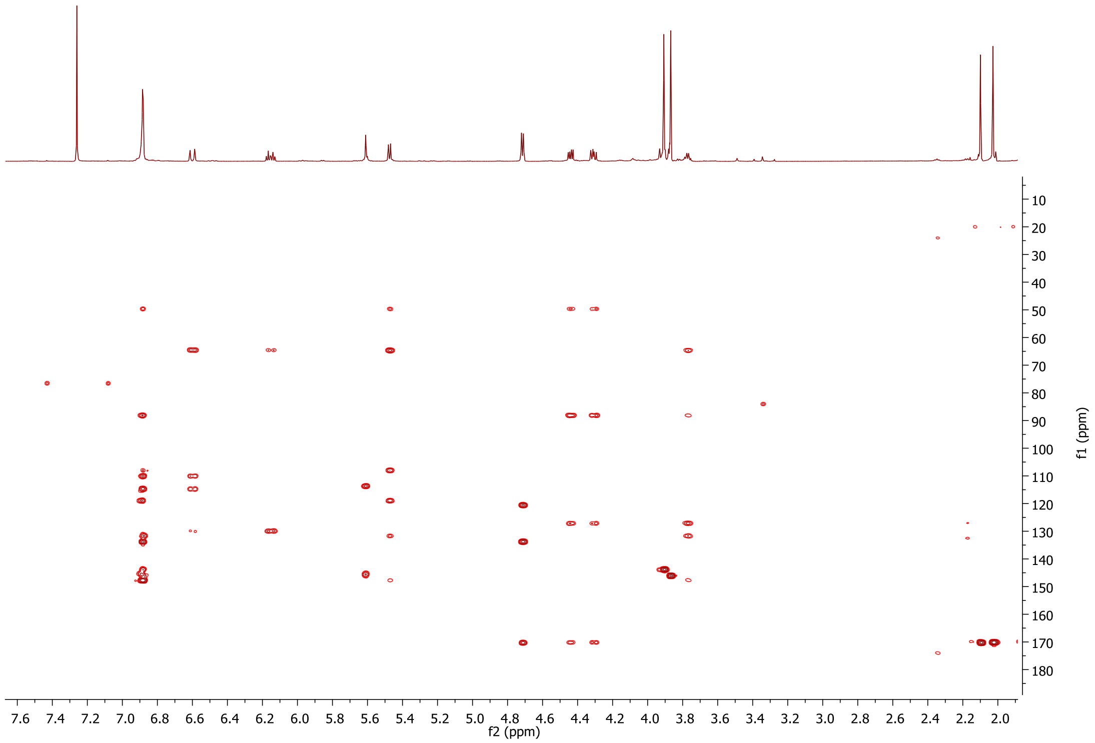

Figure S15d: HMBC spectrum of **compound 13** recorded in $CDCl_3$ at 600 MHz.

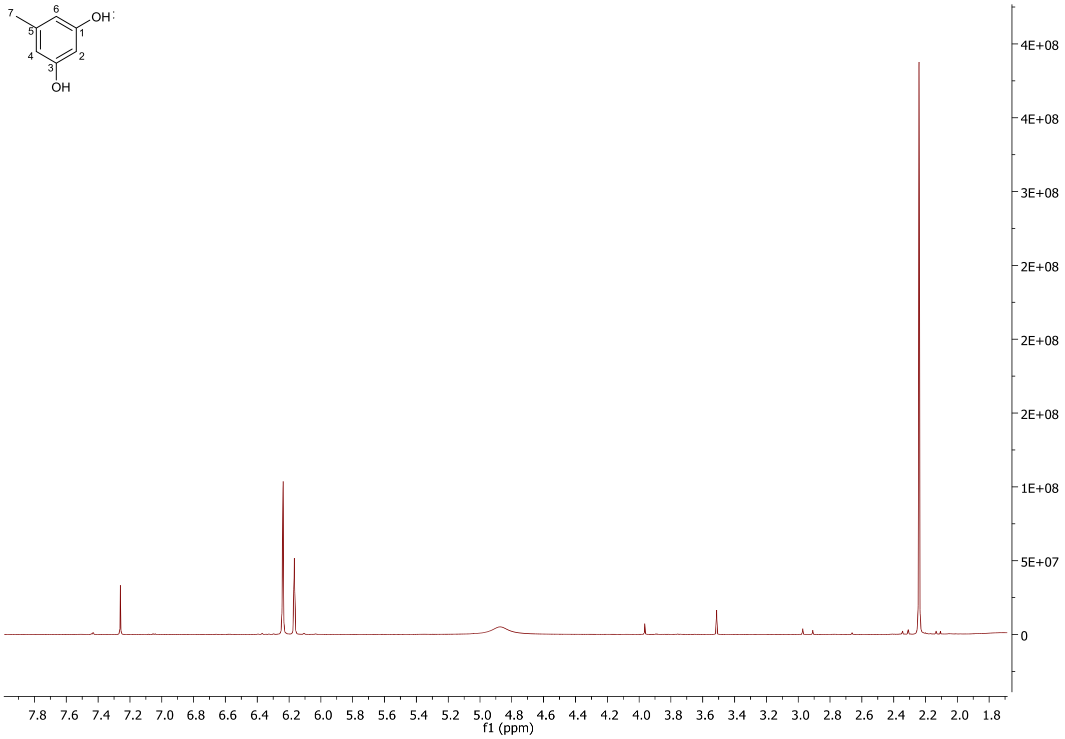

Figure S16a: ^{1}H-NMR spectrum of **compound 14** recorded in $CDCl_3$ at 600 MHz.

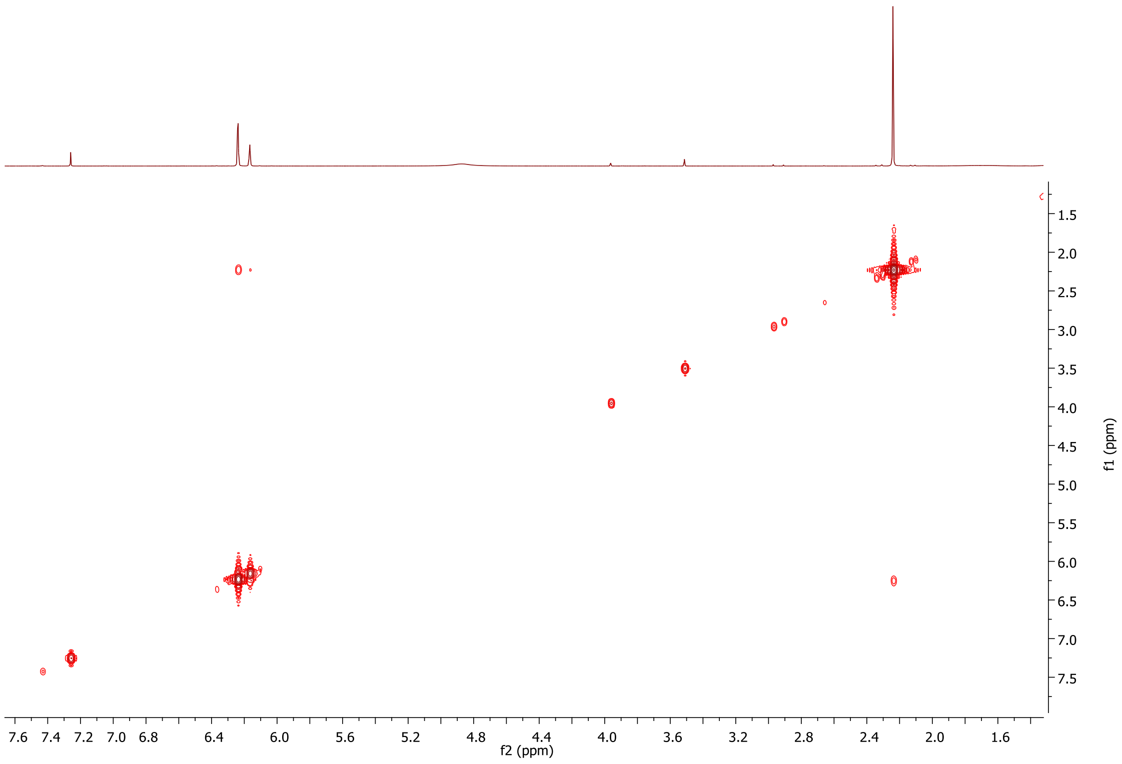

Figure S16b: COSY spectrum of **compound 14** recorded in $CDCl_3$ at 600 MHz.

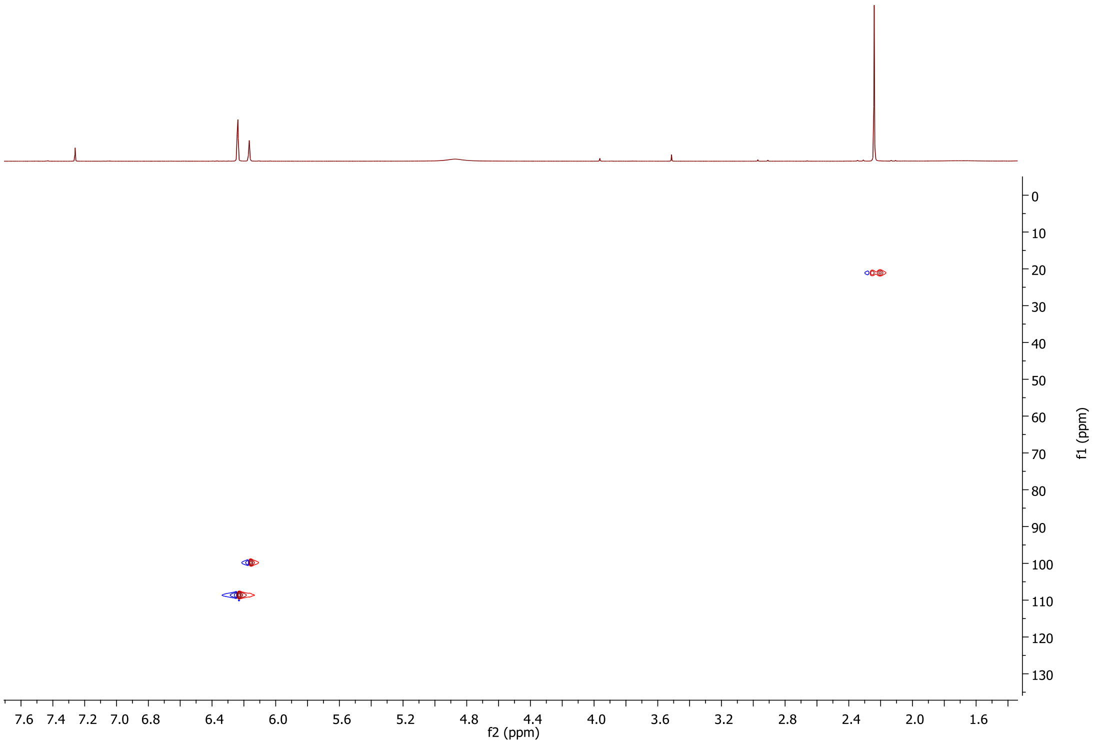

Figure S16c: HSQC-DEPT spectrum of **compound 14** recorded in $CDCl_3$ at 600 MHz.

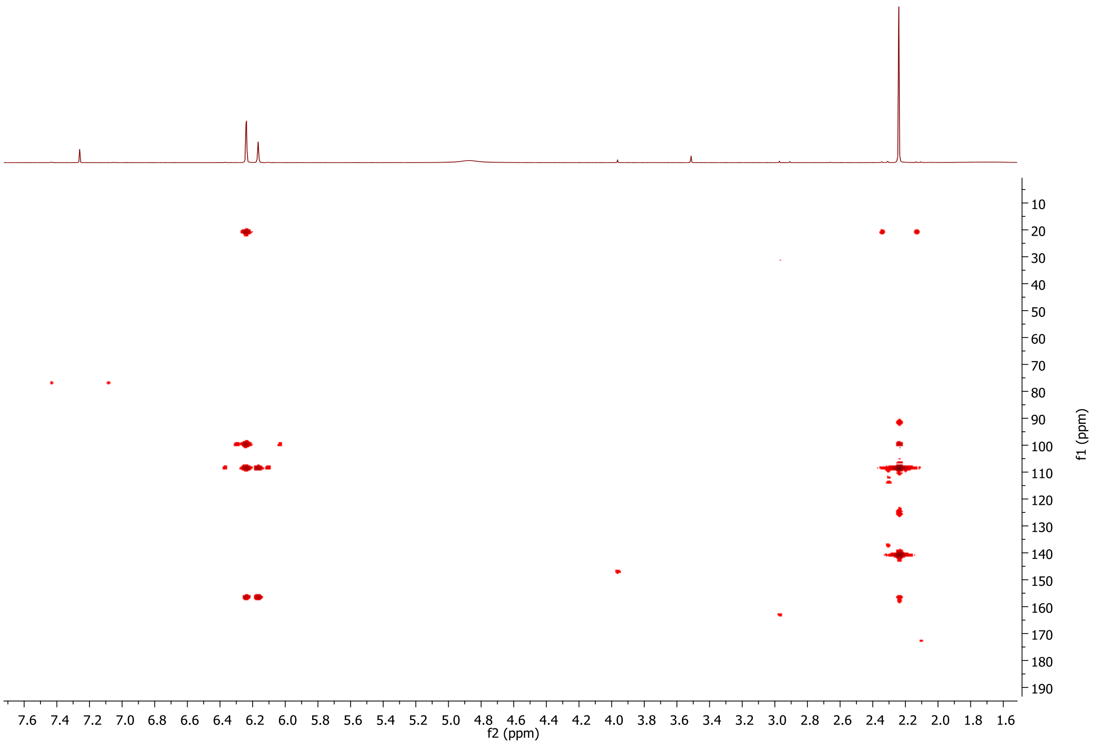

Figure S16d: HMBC spectrum of **compound 14** recorded in $CDCl_3$ at 600 MHz.

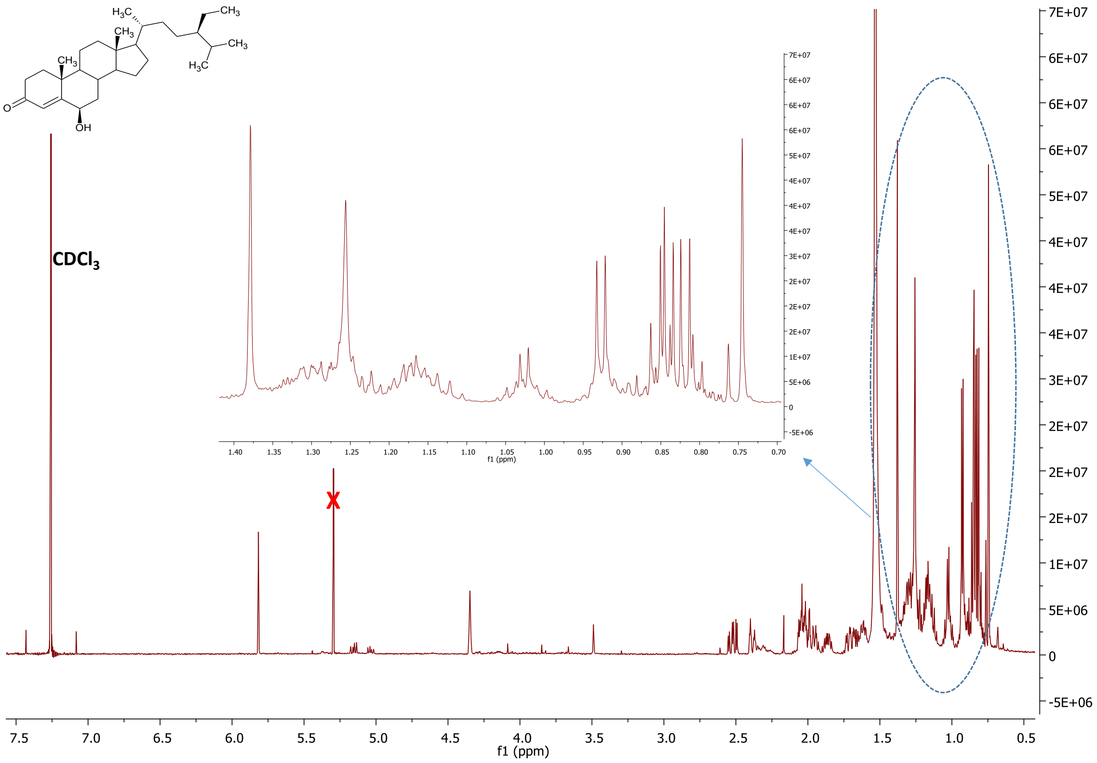

Figure S17a: ^{1}H-NMR spectrum of **compound 16** recorded in $CDCl_3$ at 600 MHz.

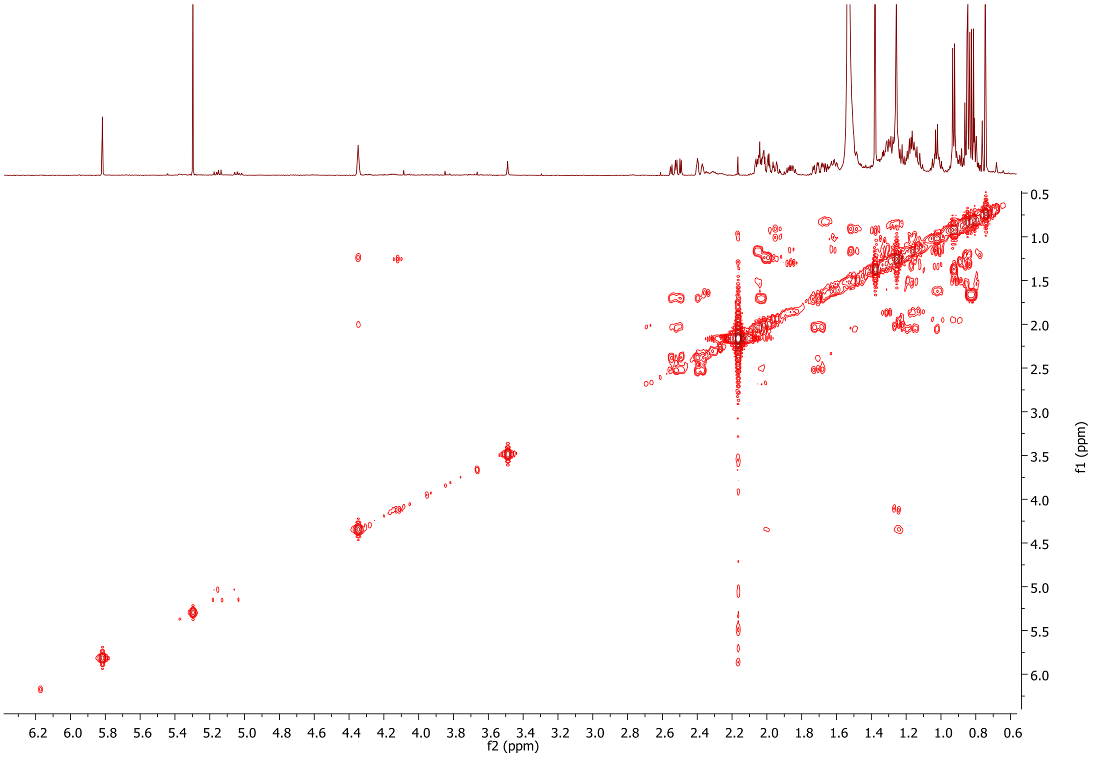

Figure S17b: COSY spectrum of **compound 16** recorded in $CDCl_3$ at 600 MHz.

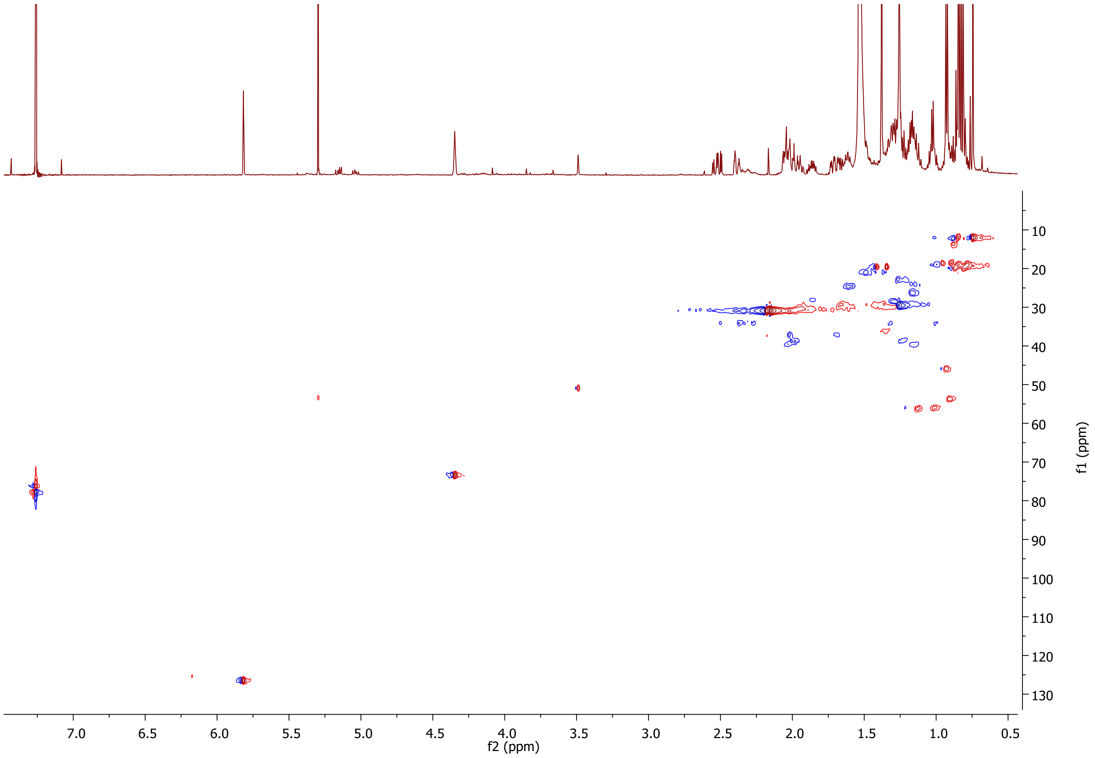

Figure S17c: HSQC-DEPT spectrum of **compound 16** recorded in $CDCl_3$ at 600 MHz.

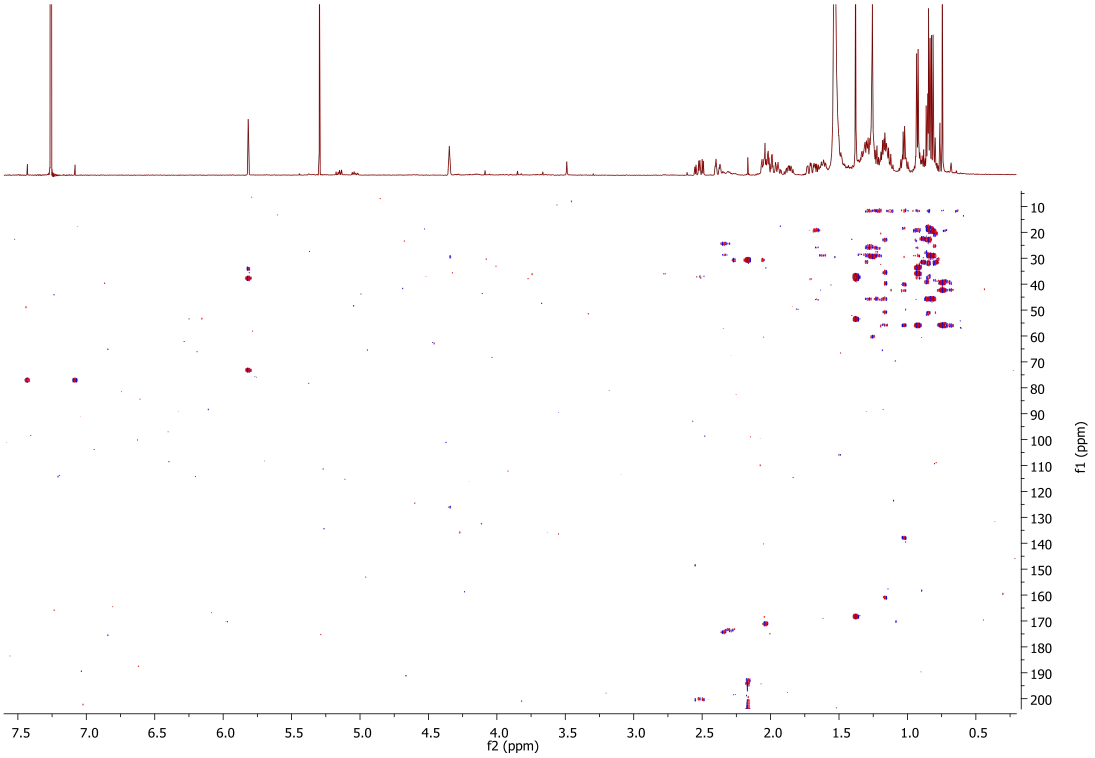

Figure S17d: HMBC spectrum of **compound 16** recorded in $CDCl_3$ at 600 MHz.
